# Dynamic analysis and optimal control considering cross transmission and variation of information

**DOI:** 10.1038/s41598-022-21774-4

**Published:** 2022-10-27

**Authors:** Sida Kang, Xilin Hou, Yuhan Hu, Hongyu Liu

**Affiliations:** 1 School of Electronic and Information Engineering, University of Science and Technology Liaoning, Anshan, 114051 China; 2 School of Science, University of Science and Technology Liaoning, Anshan, 114051 China; 3 School of Business Administration, University of Science and Technology Liaoning, Anshan, 114051 China

**Keywords:** Applied mathematics, Nonlinear phenomena

## Abstract

Cross-transmission of information has a profound influence on the progress of science and technology and the discipline integration in the field of education. In this work, knowledge gained from the viral recombination and variation in COVID-19 transmission is applied to information transmission. Virus recombination and virus variation are similar to the crossing and information fusion phenomena in information transmission. An *S*2*I*4*MR* model with information crossing and variation is constructed. Then, the local and global asymptotic stabilities of the information-free equilibrium and information-existence equilibrium are analyzed. Additionally, the basic reproduction number $R_0$ of the model is calculated. As such, an optimal control strategy is hereby proposed to promote the cross-transmission of information and generate variant information. The numerical simulations support the results of the theoretical analysis and the sensitivity of the system towards certain control parameters. In particular, the results show that strengthening information crossing promotes the generation of variant information. Furthermore, encouraging information exchange and enhancing education improve the generation of information crossing and information variation.

## Introduction

Information allows individuals to comprehend their surroundings and is critical in the development of human society. The concept of information is described in *Cybernetics* by Norbert Wiener, where he states, “Information is the content and name that human beings exchange with the external world in the process of adapting to and reacting to the external world.” Furthermore, he adds, “Including social systems, it adjusts and determines its own movements according to certain changes in the surrounding environment”^1^. The entry of new information in the social system causes fluctuations. People need to estimate the impact of information on human society to formulate strategies for promoting the information transmission that is beneficial for social development^2,3^, and at the same time suppress the information transmission that is harmful for social development^4,5^.

The ownership and transmission mode of information are the main factors that affect its process of transmission. An open social system contains a plethora of homogeneous or heterogeneous information. In addition, different types of information diffuse together and generate new information. In principle, the transmission of information is very similar to the transmission of infectious diseases^6,7^. Several literature works have adopted the classical model of infectious diseases^8–10^ to the research of rumor transmission^11^ and information transmission^12,13^. Based on this, the process of studying information transmission in the present work is inspired by the spread of *SARS-COV-2*. It is found that the mutated virus, such as the Omicron variation (B.1.1.529)^14^ has changed the transmissibility and pathogenicity of the original strain^15^. Moreover, it has also changed the way the virus impacts human society. This phenomenon of virus recombination and variation can also be applied to information cross-transmission and variation, as information also deviates from its original path during its transmission. Therefore, the model of virus recombination and variation can be employed to construct and describe the social phenomenon of information cross-transmission and variation.

The aforementioned works have presented extensive research on the cross-transmission of information. The results showed that the cross-transmission expands the scope of information transmission, and also enhances the intensity of information transmission. However, after the cross-transmission of information, new variants of information are formed, in a manner similar to a viral strain, and the mutated information generally changes the content of the original information. The natural variation of information has also been considered^27^, but so far, a limited amount of works that consider the variations caused by the cross-transmission of such information exists.

Various works presented in literature focus on the cross-transmission of information. Zan^16^ proposed *DSIR* and *C-DSIR* models and analyzed the interaction mechanism of dual rumor transmission in the BA network. The results showed that after an old rumor has been spreading for a certain period, releasing a new rumor is more favorable for the co-transmission of the two rumors. Yin et al.^17^ showed that in real social networks, multiple pieces of information exist for collaborative transmission. Based on this, a *CT*-*FSI* model was constructed to analyze the cross-transmission behavior of multiple pieces of information on Chinese microblogs and the continuous attraction index was presented. The results showed that cross-transmission of multiple pieces of information continues to spread in a cyclic manner. Yin et al.^18^ analyzed the hot topics of Chinese microblogs and observed that forwarding the information multiple times deepens the impression and spread of the topic. Therefore, the authors proposed the *MR*-*SFI* model which showed that the greater the number and intensity of re-forwarding, the wider the spread of hot topics is in microblogs. Huo et al.^19^ constructed the $I_KI_US_KS_UR$ model by considering two groups with and without scientific knowledge and analyzed the behavior of rumors spreading in each group. The results show that the group with scientific knowledge showed higher immunity to rumors. At the same time, the positive reinforcement of publicity can also resist the spread of rumors. Recently, the information transmission of *COVID-19* has attracted the focus of the research community. Yin et al.^20^ discussed that part of epidemic information is unable to reach the public in an effective and timely manner. The authors constructed the $S_1S_2F_1F_2I_{1+}I_{1-}I_2$ model, which showed that cross-transmission of information makes the transmission scope wider. In fact, information cross-transmission is pivotal in spreading rumors in a multilingual environment. By analyzing the process of rumor spreading in multiple languages in the homogeneous, heterogeneous and scale-free networks, the authors constructed the *SIR*^21^, *I*2*S*2*R*^22^, *IE*2*S*2*R*^23^, 2*I*2*SR*^24^, *IS*2*R*2^25^, *ILSR*^26^, and $S^{(1)}S^{(2)}IR$^12^ models by considering two groups. The results obtained using these models showed a common feature, i.e., that rumors spread more widely due to the cross-transmission of multiple languages, while increasing the cross-contact rate and enhancing the intensity of rumor spread.

The aforementioned works have conducted extensive research on the cross transmission of information. The results show that the cross transmission expands the scope of information transmission, and also enhances the intensity of information transmission. However, after the cross transmission of information, new variants of information are formed similar to a viral strain, and the mutated information generally changes the content of the original information. Currently, some scholars have also considered the natural variation of information^27^, but there are very few works that consider the variations caused due to the cross transmission of such information.

The modeling of cross-transmission and variation in information transmission is inspired by the phenomenon of virus recombination and variation in the COVID-19 transmission. However, information crossing and variation are desirable in certain situations. For instance, academics encourage the global integration of multi-disciplinary information and employ the new cross-disciplines in various applications. Interdisciplinary fields, such as biomathematics, physical chemistry and biochemistry, are important in the development of a wide range of applications. Therefore, the aim of this work is to propose a model that considers the cross-transmission of multiple information and the resulting variant information to determine the impact of cross-transmission of information on the resulting variant information. Furthermore, the control strategies used to enhance the intensity of information crossing and the promotion of the generation of information variants are also discussed. A system of ordinary differential equations is created to describe the problem in question. The spreading scope of information crossing on the social system is obtained by calculating the basic regeneration number. The validity of the proposed model is obtained by analyzing the equilibrium point and the stability. Finally, the basic theorem of the model and the effectiveness of the control strategy are verified by selecting appropriate parameters as the control variables and numerical simulations. In contrast to previous literature, the existence of virus recombination and variation in COVID-19 transmission is hereby compared to information transmission. Meanwhile, an information transmission model is constructed considering cross-transmission and variation, which includes multi-information and multi-transmittable groups. In addition, the optimal control strategy of information transmission is quantified by scientific methods.

The rest of this paper is organized as follows. The *S*2*I*4*MR* model that considers the information cross-transmission on social media and the generated variations is presented in "The model " Section. The local and global stability of basic reproduction number $R_0$, information-free equilibrium, and information-existence equilibrium are presented in "Stabilityanalysisofthemodel" Section. The existence and strategy for controlling the information transmission and variation in an optimal manner are presented in "The optimalcontrolmodel" Section. The influence of parameter changes on information transmission and variation and the effect of optimal control strategy based on numerical simulations are illustrated in "Numericalsimulations " Section. The sensitivity analysis of control parameters in information transmission is presented in "Sensitivityanalysis" Section. Finally, "Conclusions" Section provides the conclusion.

## The model

In this work, an open virtual community is considered. The population size is variable at any time *t*, and the total population is expressed as *N*(*t*). The population can be divided into eight categories: (1) The easy adopters who are not exposed to information but easily adopt the information, denoted as *S*(*t*); (2) People who are exposed to both kinds of information but choose to spread the first kind of information, denoted as $I_1(t)$; (3) The group exposed to both kinds of information that chooses to spread the second kind of information, denoted as $I_2(t)$; (4) The transmitters who are exposed to both kinds of information but spread the first kind of information and ultimately choose the variation group that believes in the first kind of information, denoted as $M_1(t)$; (5) The transmitters who are exposed to both kinds of information but spread the first kind of information and finally choose the variation group that integrates the two kinds of information, denoted as $M_2(t)$; (6) The transmitters who are exposed to both kinds of information but spread the second kind of information and finally choose the variation group that integrates the two kinds of information, denoted as $M_3(t)$; (7) The transmitters who are exposed to both kinds of information but spread the second kind of information and ultimately choose the variation group who believes in the second kind of information, denoted as $M_4(t)$; and (8) The fleeing crowd that is not interested in any kind of information as well as the corresponding variation information, denoted as *R*(*t*).

The model proposed in this work considers the common life phenomenon of “concept preconception”. This means that when some easy-to-adopt populations have preferential access to any information, then in their minds, the information will be transmitted first. Even if they are exposed to another kind of information, they will not transmit it immediately. This is the main differentiation between populations $I_1$ and $I_2$. At the same time, when the transmitters that are already exposed to the first kind of information are exposed to the second kind of information, they fuse the two kinds of information after a period of analysis, thus forming the variation information group. However, since they prioritize the first type of information, they will use the second kind of information as a supplement to expand the content of the first kind of information. This is the main difference between populations $M_2$ and $M_3$. Infectious disease variants generally remain transmissible. However, the information variant does not spread easily due to the uncertainty in the content of the information. This is precise because humans have subjective judgments, whereas viruses are generally a result of natural selection.

**Figure 1:**
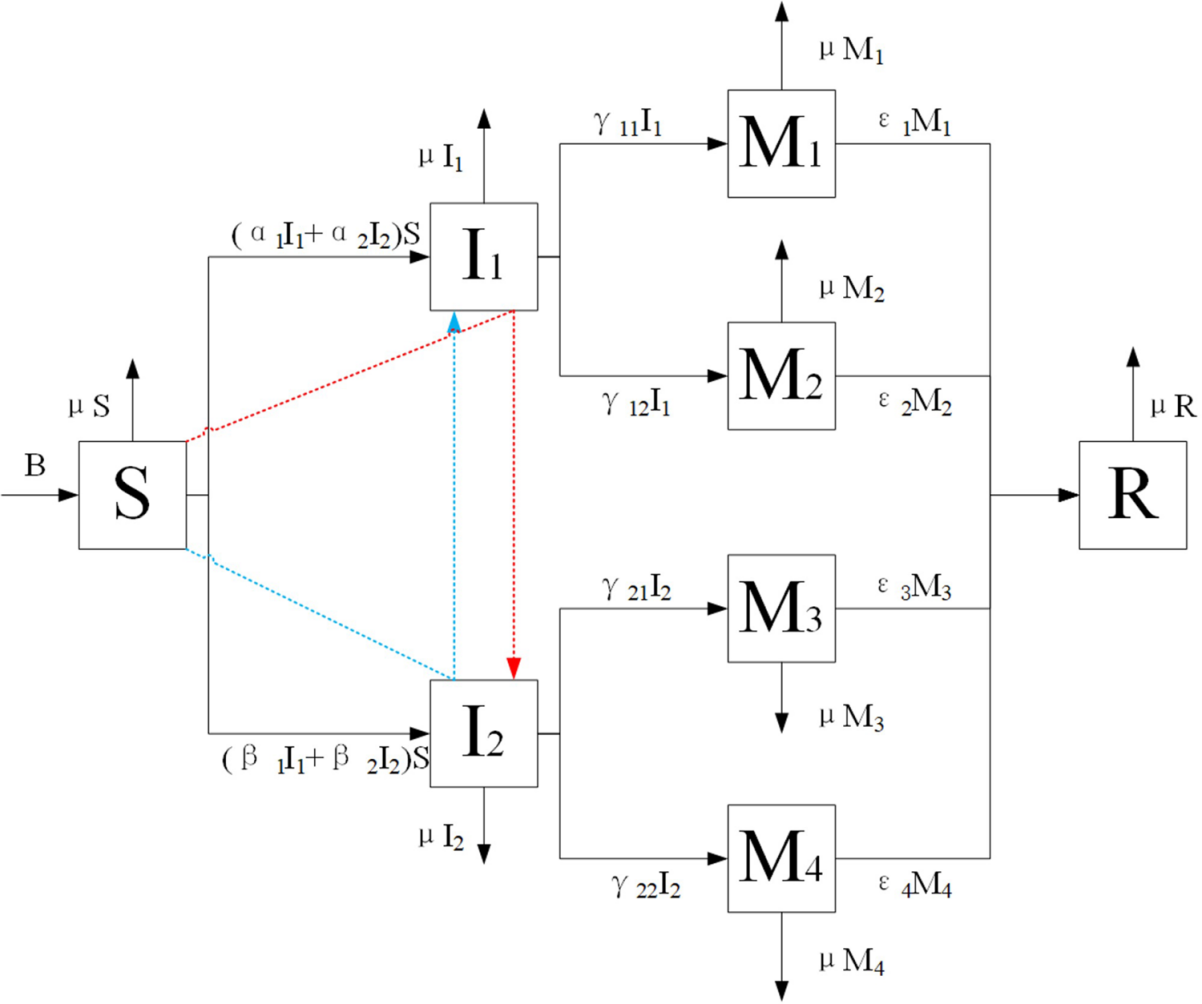
The flow diagram of the model.

In order to reflect the phenomenon of cross transmission and information variation in information transmission, an *S*2*I*4*MR* model is constructed in this work. The model flow diagram is given in Fig. 1.

The parameters of *S*2*I*4*MR* model are interpreted as follows:

- In a social system, the number of individuals generally varies over time. Therefore, this work defines *B* as the number of immigrants in the social system. At the same time, it considers some individuals that may withdraw from the social system due to some force majeure factors. $\mu$ is defined as the emigration rate in this work;
- When information begins to spread in a social system, there is a certain progression rate that the easy adopters will contact the transmitters of the information. When the easy adopters are first exposed to the first kind of information and then to the second kind, they prioritize spreading the first kind of information. The contact rate of the first information is defined as $\alpha _1$, and the contact rate of the second information as $\alpha _2$. In order to express the phenomenon of “concept preconception”, here, $\alpha _1\ge \alpha _2$, and the group exposed to the first kind of information transmitters with the progression rate of $\alpha _1$ must include the group exposed to the second kind of information transmitters with progression rate of $\alpha _2$. In the same way, for the group that preferentially transmits the second kind of information, the contact rate of the first kind of information is $\beta _1$, and the contact rate of the second kind of information is $\beta _2$. In order to represent the phenomenon of “concept preconception”, $\beta _1\le \beta _2$, and the group exposed to the second kind of information transmitters with the progression rate of $\beta _2$ must include the group exposed to the first transmitters with the progression rate of $\beta _1$;
- When the transmitters of two kinds of information fuse the information, the information variation is generated. When the transmitters of both types of information believe in the original information, they are mutated into $M_1$ and $M_4$ groups with the progression rate of $\gamma _{11}$ and $\gamma _{22}$, respectively. When the transmitters of the two kinds of information choose to fuse the information, they mutate with the progression rate of $\gamma _{12}$ into the $M_2$ group that regards the first kind of information as the main and the second kind of information as the auxiliary. The transmitters mutate with the progression rate of $\gamma _{21}$ into the $M_3$ group that considers the second kind of information as the main and the first kind of information as the auxiliary;
- After the information exists the social system after a certain period of time, it is often eliminated by the society, or is no longer accepted by people. Therefore, the variation group chooses to escape with the progression rate of $\varepsilon _1$, $\varepsilon _2$, $\varepsilon _3$, and $\varepsilon _4$.

Based on the aforementioned analysis, we constructed the *S*2*I*4*MR* model by considering the cross transmission and variation of information. In order to facilitate the understanding and analysis, a special case is considered where the easy-adopter populations are exposed to both kinds of information with equal progression rate, i.e., $\alpha _1=\alpha _2=\alpha$ and $\beta _1=\beta _2=\beta$. Therefore, the fraction of the population as proceeding form from *S* to $I_1$ is $\alpha$. In addition, the fraction of the population as proceeding form from *S* to $I_2$ is $\beta$.

The parameters of *S*2*I*4*MR* model are summarized in Table 1.

**Table 1:** The parameters description of *S*2*I*4*MR* model.

Parameter	Description
*S*(*t*)	The number of easy adopters at the time *t*
$I_1(t)$	The number of individuals choose to spread the first kind of information at the time *t*
$I_2(t)$	The number of individuals choose to spread the second kind of information at the time *t*
$M_1(t)$	The transmitters who ultimately choose the variation group that believes in the first kind of information
	at the time *t*
$M_2(t)$	The transmitters who finally choose the variation group that integrates the two kinds of information at the time *t*
$M_3(t)$	The transmitters who finally choose the variation group that integrates the two kinds of information at the time *t*
$M_4(t)$	The transmitters who ultimately choose the variation group that believes in the second kind of information
	at the time *t*
*R*(*t*)	The number of individuals that not interested in both kinds of information as well as the corresponding
	variation information at the time *t*
*B*	The number of immigrants in the social system per unit time
$\alpha$	Progression rate from state *S* to $I_1$
$\beta$	Progression rate from state *S* to $I_2$
$\gamma _{11}$	Progression rate from state $I_1$ to $M_1$
$\gamma _{12}$	Progression rate from state $I_1$ to $M_2$
$\gamma _{21}$	Progression rate from state $I_2$ to $M_3$
$\gamma _{22}$	Progression rate from state $I_2$ to $M_4$
$\varepsilon _1$,$\varepsilon _2$,$\varepsilon _3$,$\varepsilon _4$	Progression rate from state $M_1$, $M_2$, $M_3$, $M_4$ to *R*
$\mu$	Removal rate per unit time

The system dynamics are mathematically expressed as follows:

1 $$\left\{ {\begin{array}{*{20}l}    {\frac{{dS}}{{dt}}} \hfill &  =  \hfill & {B - \alpha (I_{1}  + I_{2} )S - \beta (I_{1}  + I_{2} )S - \mu S,} \hfill  \\    {\frac{{dI_{1} }}{{dt}}} \hfill &  =  \hfill & {\alpha (I_{1}  + I_{2} )S - \gamma _{{11}} I_{1}  - \gamma _{{12}} I_{1}  - \mu I_{1} ,} \hfill  \\    {\frac{{dI_{2} }}{{dt}}} \hfill &  =  \hfill & {\beta (I_{1}  + I_{2} )S - \gamma _{{22}} I_{2}  - \gamma _{{21}} I_{2}  - \mu I_{2} ,} \hfill  \\    {\frac{{dM_{1} }}{{dt}}} \hfill &  =  \hfill & {\gamma _{{11}} I_{1}  - \varepsilon _{1} M_{1}  - \mu M_{1} ,} \hfill  \\    {\frac{{dM_{2} }}{{dt}}} \hfill &  =  \hfill & {\gamma _{{12}} I_{1}  - \varepsilon _{2} M_{2}  - \mu M_{2} ,} \hfill  \\    {\frac{{dM_{3} }}{{dt}}} \hfill &  =  \hfill & {\gamma _{{21}} I_{2}  - \varepsilon _{3} M_{3}  - \mu M_{3} ,} \hfill  \\    {\frac{{dM_{4} }}{{dt}}} \hfill &  =  \hfill & {\gamma _{{22}} I_{2}  - \varepsilon _{4} M_{4}  - \mu M_{4} ,} \hfill  \\    {\frac{{dR}}{{dt}}} \hfill &  =  \hfill & {\varepsilon _{1} M_{1}  + \varepsilon _{2} M_{2}  + \varepsilon _{3} M_{3}  + \varepsilon _{4} M_{4}  - \mu R.} \hfill  \\   \end{array} } \right. $$

Where:

2 $$\begin{aligned} \begin{aligned} \ \begin{array}{l} B> 0,\mu> 0,{\varepsilon _1}> 0,{\varepsilon _2}> 0,{\varepsilon _3}> 0,{\varepsilon _4} > 0,\\ \alpha \in (0,1],\beta \in (0,1],{\gamma _{11}} \in (0,1],{\gamma _{12}} \in (0,1],{\lambda _{21}} \in (0,1],{\gamma _{22}} \in (0,1], \end{array}\ \end{aligned} \end{aligned}$$

and

3 $$\begin{aligned} \begin{aligned} \ S(t) + {I_1}(t) + {I_2}(t) + {M_1}(t) + {M_2}(t) + {M_3}(t) + {M_4}(t) + R(t) = N(t).\ \end{aligned} \end{aligned}$$

It is easy to know that $\frac{{dN(t)}}{{dt}} = B - \mu N$, so $N(t) = \left( {N_0} - \frac{B}{\mu }\right) {e^{ - \mu t}} + \frac{B}{\mu }$, where $N_0=N(0)$, and then ${\lim _{t \rightarrow \infty }}N(t) = \frac{B}{\mu }$. The positive invariant set of System (1) is $\Gamma = \left\{ {\left( {S,{I_1},{I_2},{M_1},{M_2},{M_3},{M_4},R} \right) \in R_8^ + :S + {I_1} + {I_2} + {M_1} + {M_2} + {M_3} + {M_4} + R \le \frac{B}{\mu }} \right\}$.

## Stability analysis of the model

Firstly, it is necessary to demonstrate the existence of equilibrium $E = (S,{I_1},{I_2},{M_1},{M_2},{M_3},{M_4},R)$ of the system dynamics Eq. (1). The information-free equilibrium point of System (1) can be easily obtained as ${E^0} = ({{B} \!{/} \!{\mu }},0,0,0,0,0,0,0)$, which means the number of information disseminators tend to zero in System (1).

Then, the basic reproduction number $R_0$ of System (1) can be defined by the next generation matrix^28^. The basic reproduction number is important to intervene for a system, which represents the number of next generation from a single information disseminator produced.

Let $X = {({I_1},{I_2},S,{M_1},{M_2},{M_3},{M_4},R)^T}$, then System (1) can be written as:

4 $$\frac{{dX}}{{dt}} = F(X) - V(X)$$

5 $$\begin{aligned} & \begin{aligned} \ F(X) = \left( {\begin{array}{*{20}{c}} {\alpha ({I_1} + {I_2})S}\\ {\beta ({I_1} + {I_2})S}\\ 0\\ 0\\ 0\\ 0\\ 0\\ 0 \end{array}} \right) ,V(X) = \left( {\begin{array}{*{20}{l}} {{\gamma _{11}}{I_1} + {\gamma _{12}}{I_1} + \mu {I_1}}\\ {{\gamma _{22}}{I_2} + {\gamma _{21}}{I_2} + \mu {I_2}}\\ { - B + \alpha ({I_1} + {I_2})S + \beta ({I_1} + {I_2})S + \mu S}\\ { - {\gamma _{11}}{I_1} + {\varepsilon _1}{M_1} + \mu {M_1}}\\ { - {\gamma _{12}}{I_1} + {\varepsilon _2}{M_2} + \mu {M_2}}\\ { - {\gamma _{21}}{I_2} + {\varepsilon _3}{M_3} + \mu {M_3}}\\ { - {\gamma _{22}}{I_2} + {\varepsilon _4}{M_4} + \mu {M_4}}\\ { - {\varepsilon _1}{M_1} - {\varepsilon _2}{M_2} - {\varepsilon _3}{M_3} - {\varepsilon _4}{M_4} + \mu R} \end{array}} \right) .\ \end{aligned} \end{aligned}$$

We can get:

6 $$\begin{aligned} \begin{aligned} \ F = \left( {\begin{array}{*{20}{c}} {\alpha S}&{}{\alpha S}\\ {\beta S}&{}{\beta S} \end{array}} \right) ,V = \left( {\begin{array}{*{20}{c}} {{\gamma _{11}} + {\gamma _{12}} + \mu }&{}0\\ 0&{}{{\gamma _{22}} + {\gamma _{21}} + \mu } \end{array}} \right) .\ \end{aligned} \end{aligned}$$

where *F* and *V* represent the infection and transition matrices respectively^29^. Hence, the basic reproduction number $R_0$ of System (1) is the spectral radius of the next generation matrix $F{V^ {-1} }$. $R_0$ can be computed as:

7 $$\begin{aligned} \begin{aligned} \ {R_0} = \rho (F{V^{ - 1}}) = \frac{{B\alpha ({\gamma _{22}} + {\gamma _{21}} + \mu ) + B\beta ({\gamma _{11}} + {\gamma _{12}} + \mu )}}{{\mu ({\gamma _{11}} + {\gamma _{12}} + \mu )({\gamma _{22}} + {\gamma _{21}} + \mu )}}.\ \end{aligned} \end{aligned}$$

While the information will be spread if $R_0>1$. The information-existence equilibrium point of System (1) can be expressed as ${E^*} = ({S^*},I_1^*,I_2^*,M_1^*,M_2^*,M_3^*,M_4^*,{R^*})$, which means the information will spread widely. The information-existence equilibrium ${E^*}$ should satisfy:

8 $$\begin{aligned} \begin{aligned} \ \left\{ {\begin{array}{*{20}{l}} {B - \alpha ({I_1}^* + {I_2}^*)S^* - \beta ({I_1}^* + {I_2}^*)S^* - \mu S^*}&{} = &{}{0,}\\ {\alpha ({I_1}^* + {I_2}^*)S^* - {\gamma _{11}}{I_1}^* - {\gamma _{12}}{I_1}^* - \mu {I_1}^*}&{} = &{}{0,}\\ {\beta ({I_1}^* + {I_2}^*)S^* - {\gamma _{22}}{I_2}^* - {\gamma _{21}}{I_2}^* - \mu {I_2}^*}&{} = &{}{0,}\\ {{\gamma _{11}}{I_1}^* - {\varepsilon _1}{M_1}^* - \mu {M_1}^*}&{} = &{}{0,}\\ {{\gamma _{12}}{I_1}^* - {\varepsilon _2}{M_2}^* - \mu {M_2}^*}&{} = &{}{0,}\\ {{\gamma _{21}}{I_2}^* - {\varepsilon _3}{M_3}^* - \mu {M_3}^*}&{} = &{}{0,}\\ {{\gamma _{22}}{I_2}^* - {\varepsilon _4}{M_4}^* - \mu {M_4}^*}&{} = &{}{0,}\\ {{\varepsilon _1}{M_1}^* + {\varepsilon _2}{M_2}^* + {\varepsilon _3}{M_3}^* + {\varepsilon _4}{M_4}^* - \mu R^*}&{} = &{}{0.} \end{array}} \right. \ \end{aligned} \end{aligned}$$

Let $\varphi = I_1^* + I_2^*$. The information-existence equilibrium ${E^*}$ can be deduced as the following equations by solving Eqs. (8):

9 $$\begin{aligned} \begin{aligned} \ {S^*} = \frac{B}{{\alpha \varphi + \beta \varphi + \mu }},I_1^* = \frac{{\alpha \varphi {S^*}}}{{({\gamma _{11}} + {\gamma _{12}} + \mu )}},I_2^* = \frac{{\beta \varphi {S^*}}}{{({\gamma _{22}} + {\gamma _{21}} + \mu )}}.\ \end{aligned} \end{aligned}$$

Since

10 $$\begin{aligned} \begin{aligned} \ \varphi = {I_1} + {I_2} = \frac{{\alpha \varphi {S^*}}}{{({\gamma _{11}} + {\gamma _{12}} + \mu )}} + \frac{{\beta \varphi {S^*}}}{{({\gamma _{22}} + {\gamma _{21}} + \mu )}},\ \end{aligned} \end{aligned}$$

we can get

11 $$\begin{aligned} \begin{aligned} \ {S^*} = \frac{{({\gamma _{11}} + {\gamma _{12}} + \mu )({\gamma _{22}} + {\gamma _{21}} + \mu )}}{{\alpha ({\gamma _{22}} + {\gamma _{21}} + \mu ) + \beta ({\gamma _{11}} + {\gamma _{12}} + \mu )}} = \frac{B}{{\mu {R_0}}},\ \end{aligned} \end{aligned}$$

and

12 $$\begin{aligned} \begin{aligned} \ \varphi = \frac{{\mu ({R_0} - 1)}}{{\alpha + \beta }},\ \end{aligned} \end{aligned}$$

the other equilibrium points can be obtained as:

13 $$\begin{aligned} \begin{aligned} \ \begin{array}{l} I_1^* = \frac{{B\alpha \varphi }}{{\mu ({\gamma _{11}} + {\gamma _{12}} + \mu ){R_0}}},I_2^* = \frac{{B\beta \varphi }}{{\mu ({\gamma _{22}} + {\gamma _{21}} + \mu ){R_0}}},\\ M_1^* = \frac{{B\alpha {\gamma _{11}}\varphi }}{{\mu ({\varepsilon _1} + \mu )({\gamma _{11}} + {\gamma _{12}} + \mu ){R_0}}},M_2^* = \frac{{B\alpha {\gamma _{12}}\varphi }}{{\mu ({\varepsilon _2} + \mu )({\gamma _{11}} + {\gamma _{12}} + \mu ){R_0}}},\\ M_3^* = \frac{{B\beta {\gamma _{21}}\varphi }}{{\mu ({\varepsilon _3} + \mu )({\gamma _{22}} + {\gamma _{21}} + \mu ){R_0}}},M_4^* = \frac{{B\beta {\gamma _{22}}\varphi }}{{\mu ({\varepsilon _4} + \mu )({\gamma _{22}} + {\gamma _{21}} + \mu ){R_0}}}. \end{array}\ \end{aligned} \end{aligned}$$

### Theorem 1

If $R_0<1$, $B\left( {\alpha + \beta } \right) < \mu ({\gamma _{11}} + {\gamma _{12}} + \mu )({\gamma _{22}} + {\gamma _{21}} + \mu )$ and $({\gamma _{11}} + {\gamma _{12}} + \mu ) = ({\gamma _{22}} + {\gamma _{21}} + \mu )$, the information-free equilibrium point ${E^0} = ({{B} \!{/} \!{\mu }},0,0,0,0,0,0,0)$ of System (1) is locally asymptotically stable.

### Proof 1

The Jacobin matrix of System (1) at information-free equilibrium point ${E^0} = ({{B} \!{/} \!{\mu }},0,0,0,0,0,0,0)$ can be written as:

14 $$\begin{aligned} \begin{aligned} { \ J({E^0}) = \left[ {\begin{array}{*{20}{c}} { - \mu }&{}{ - \frac{{B\alpha }}{\mu } - \frac{{B\beta }}{\mu }}&{}{ - \frac{{B\alpha }}{\mu } - \frac{{B\beta }}{\mu }}&{}0&{}0&{}0&{}0&{}0\\ 0&{}{\frac{{B\alpha }}{\mu } - ({\gamma _{11}} + {\gamma _{12}} + \mu )}&{}{\frac{{B\alpha }}{\mu }}&{}0&{}0&{}0&{}0&{}0\\ 0&{}{\frac{{B\beta }}{\mu }}&{}{\frac{{B\beta }}{\mu } - ({\gamma _{22}} + {\gamma _{21}} + \mu )}&{}0&{}0&{}0&{}0&{}0\\ 0&{}{{\gamma _{11}}}&{}0&{}{ - {\varepsilon _1} - \mu }&{}0&{}0&{}0&{}0\\ 0&{}{{\gamma _{12}}}&{}0&{}0&{}{ - {\varepsilon _2} - \mu }&{}0&{}0&{}0\\ 0&{}0&{}{{\gamma _{21}}}&{}0&{}0&{}{ - {\varepsilon _3} - \mu }&{}0&{}0\\ 0&{}0&{}{{\gamma _{22}}}&{}0&{}0&{}0&{}{ - {\varepsilon _4} - \mu }&{}0\\ 0&{}0&{}0&{}0&{}0&{}0&{}0&{}{ - \mu } \end{array}} \right] .\ } \end{aligned} \end{aligned}$$

The negative eigenvalues of $J({E^0})$ can be easily obtained as $${\Lambda _{01}} = {\Lambda _{02}} = - \mu< 0,{\Lambda _{03}} = - {\varepsilon _1} - \mu< 0,{\Lambda _{04}} = - {\varepsilon _2} - \mu< 0,{\Lambda _{05}} = - {\varepsilon _3} - \mu< 0,{\Lambda _{06}} = - {\varepsilon _4} - \mu < 0\ $$, and the other eigenvalues are the characteristic roots of $\ \left| {hE - J({E^0})} \right|$, where:

15 $$\begin{aligned} \begin{aligned} \ \left| {hE - J({E^0})} \right| = \left| {\begin{array}{*{20}{c}} {h - \frac{{B\alpha }}{\mu } + ({\gamma _{11}} + {\gamma _{12}} + \mu )}&{}{ - \frac{{B\alpha }}{\mu }}\\ { - \frac{{B\beta }}{\mu }}&{}{h - \frac{{B\beta }}{\mu } + ({\gamma _{22}} + {\gamma _{21}} + \mu )} \end{array}} \right| .\ \end{aligned} \end{aligned}$$

The eigenvalues of Eq. (15) can be obviously obtained as:

16 $$\begin{aligned} {{\Lambda _{07}}}&= {\frac{{\left[ {\frac{{B\alpha }}{\mu } + \frac{{B\beta }}{\mu } - ({\gamma _{11}} + {\gamma _{12}} + \mu ) - ({\gamma _{22}} + {\gamma _{21}} + \mu )} \right] }}{2}}\\ \nonumber&\quad + {\frac{{\sqrt{{{\left[ {\frac{{B\alpha }}{\mu } + \frac{{B\beta }}{\mu } - ({\gamma _{11}} + {\gamma _{12}} + \mu ) - ({\gamma _{22}} + {\gamma _{21}} + \mu )} \right] }^2} + 4({\gamma _{11}} + {\gamma _{12}} + \mu )({\gamma _{22}} + {\gamma _{21}} + \mu )({R_0} - 1)} }}{2}}, \end{aligned}$$

and

17 $$\begin{aligned} {{\Lambda _{08}}}&= {\frac{{\left[ {\frac{{B\alpha }}{\mu } + \frac{{B\beta }}{\mu } - ({\gamma _{11}} + {\gamma _{12}} + \mu ) - ({\gamma _{22}} + {\gamma _{21}} + \mu )} \right] }}{2}}\\ \nonumber {}&\quad - {\frac{{\sqrt{{{\left[ {\frac{{B\alpha }}{\mu } + \frac{{B\beta }}{\mu } - ({\gamma _{11}} + {\gamma _{12}} + \mu ) - ({\gamma _{22}} + {\gamma _{21}} + \mu )} \right] }^2} + 4({\gamma _{11}} + {\gamma _{12}} + \mu )({\gamma _{22}} + {\gamma _{21}} + \mu )({R_0} - 1)} }}{2}}. \end{aligned}$$

If $B\left( {\alpha + \beta } \right) < \mu ({\gamma _{11}} + {\gamma _{12}} + \mu )({\gamma _{22}} + {\gamma _{21}} + \mu )$ and $({\gamma _{11}} + {\gamma _{12}} + \mu ) = ({\gamma _{22}} + {\gamma _{21}} + \mu )$, so ${\Lambda _{07}}<0$ and ${\Lambda _{08}}<0$. Hence, the information-free equilibrium point ${E^0} = ({{B} \!{/} \!{\mu }},0,0,0,0,0,0,0)$ of System (1) is locally asymptotically stable based on the Routh–Hurwitz criterion. $\square$

### Theorem 2

If $R_0<1$ and $B\left( {\alpha + \beta } \right) \le {\mu ^2}$, the information-free equilibrium point ${E^0} = ({{B} \!{/} \!{\mu }},0,0,0,0,0,0,0)$ of System (1) is globally asymptotically stable.

### Proof 2

It is easy to know that $S(t) + {I_1}(t) + {I_2}(t) + {M_1}(t)+ {M_2}(t)+ {M_3}(t)+ {M_4}(t) + R(t) = N(t)$ and satisfy $\frac{{dN(t)}}{{dt}} \le B - \mu S(t)$. It illustrates that:

18 $$\begin{aligned} \begin{aligned} \ \mathop {\lim }\limits _{t \rightarrow 0} \sup N(t) \le \frac{B}{\mu }.\ \end{aligned} \end{aligned}$$

For $t \ge 0$, the positive invariant set of System (1) can be written as:

19 $$\begin{aligned} \begin{aligned} \ \begin{array}{*{20}{l}} T&{} = &{}{\{ (S(t),{I_1}(t),{I_2}(t),{M_1}(t),{M_2}(t),{M_3}(t),{M_4}(t),R(t)) \in R_8^ +:}\\ {}&{}{}&{}{S(t) + {I_1}(t) + {I_2}(t) + {M_1}(t) + {M_2}(t) + {M_3}(t) + {M_4}(t) + R(t) \le \frac{B}{\mu }\}.} \end{array}\ \end{aligned} \end{aligned}$$

Then, the Lyapunov function $L(t) = {I_1}(t) + {I_2}(t) + {M_1}(t)+ {M_2}(t)+ {M_3}(t)+ {M_4}(t) + R(t)$ can be constructed and $L'(t)$ can be computed as:

20 $$\begin{aligned} {L'(t)}&= {\alpha ({I_1} + {I_2})S - {\gamma _{11}}{I_1} - {\gamma _{12}}{I_1} - \mu {I_1} + \beta ({I_1} + {I_2})S - {\gamma _{22}}{I_2} - {\gamma _{21}}{I_2}}\\\nonumber {}&\quad - {\mu {I_2} + {\gamma _{11}}{I_1} - {\varepsilon _1}{M_1} - \mu {M_1} + {\gamma _{12}}{I_1} - {\varepsilon _2}{M_2} - \mu {M_2} + {\gamma _{21}}{I_2} - {\varepsilon _3}{M_3}}\\ \nonumber {}&\quad - {\mu {M_3} + {\gamma _{22}}{I_1} - {\varepsilon _4}{M_4} - \mu {M_4} + {\varepsilon _1}{M_1} + {\varepsilon _2}{M_2} + {\varepsilon _3}{M_3} + {\varepsilon _4}{M_4} - \mu R}\\\nonumber {}&={( - \mu + \alpha S + \beta S)({I_1} + {I_2}) - \mu {M_1} - \mu {M_2} - \mu {M_3} - \mu {M_4} - \mu R}\\ \nonumber {}&\le {\left( - \mu + \frac{{B\alpha }}{\mu } + \frac{{B\beta }}{\mu }\right) ({I_1} + {I_2}) - \mu ({M_1} + {M_2} + {M_3} + {M_4} + R)}, \end{aligned}$$

it is easy to know that $L'(t) \le 0$ if $S \le \frac{B}{\mu }$ and $B\left( {\alpha + \beta } \right) \le {\mu ^2}$.

In addition, $L'(t) = 0$ holds if and only if $S(t) = {S^0},{I_1}= {I_2}= {M_1}= {M_2}= {M_3}= {M_4}=R=0$. From System (1), it is known that ${E^0}$ is the only solution in *T* when $L'(t) = 0$. Therefore, based on the Lyapunov-LaSalle Invariance Principle^30^, it is shown that every solution of System (1) approach ${E^0}$ for $t \rightarrow \infty$. Hence, the information-free equilibrium point ${E^0} = ({{B} \!{/}\!{\mu }},0,0,0,0,0,0,0)$ of System (1) is globally asymptotically stable. $\square$

### Theorem 3

If $R_0>1$ and ${\mu ^2}{R_0}({R_0}-1) > B(\alpha + \beta )$, the information-existence equilibrium point ${E^*} = ({S^*},I_1^*,I_2^*,M_1^*,M_2^*,M_3^*, M_4^*,{R^*})$ of system (1) is locally asymptotically stable.

### Proof 3

The Jacobin matrix of System (1) at information-existence equilibrium point ${E^*} = ({S^*},I_1^*,I_2^*,M_1^*,M_2^*,M_3^*,M_4^*,{R^*})$ can be written as:

21 $$\begin{aligned} \begin{aligned} { \ J({E^*}) = \left[ {\begin{array}{*{20}{c}} { \!-\! \alpha {\varphi ^*} \!-\! \beta {\varphi ^*} \!-\! \mu }&{}{ \!-\! \alpha {S^*} \!-\! \beta {S^*}}&{}{ \!-\! \alpha {S^*} \!-\! \beta {S^*}}&{}0&{}0&{}0&{}0&{}0\\ {\alpha {\varphi ^*}}&{}{\alpha {S^*} \!-\! ({\gamma _{11}} \!+\! {\gamma _{12}} \!+\! \mu )}&{}{\alpha {S^*}}&{}0&{}0&{}0&{}0&{}0\\ {\beta {\varphi ^*}}&{}{\beta {S^*}}&{}{\beta {S^*} \!-\! ({\gamma _{22}} \!+\! {\gamma _{21}} \!+\! \mu )}&{}0&{}0&{}0&{}0&{}0\\ 0&{}{{\gamma _{11}}}&{}0&{}{ \!-\! {\varepsilon _1} \!-\! \mu }&{}0&{}0&{}0&{}0\\ 0&{}{{\gamma _{12}}}&{}0&{}0&{}{ \!-\! {\varepsilon _2} \!-\! \mu }&{}0&{}0&{}0\\ 0&{}0&{}{{\gamma _{21}}}&{}0&{}0&{}{ \!-\! {\varepsilon _3} \!-\! \mu }&{}0&{}0\\ 0&{}0&{}{{\gamma _{22}}}&{}0&{}0&{}0&{}{ \!-\! {\varepsilon _4} \!-\! \mu }&{}0\\ 0&{}0&{}0&{}0&{}0&{}0&{}0&{}{ \!-\! \mu } \end{array}} \right] .\ } \end{aligned} \end{aligned}$$

The negative eigenvalues of $J({E^*})$ can be easily obtained as $${\Lambda _{11}} = - \mu< 0,{\Lambda _{12}} = - {\varepsilon _1} - \mu< 0,{\Lambda _{13}} = - {\varepsilon _2} - \mu< 0,{\Lambda _{14}} = - {\varepsilon _3} - \mu< 0,{\Lambda _{15}} = - {\varepsilon _4} - \mu < 0\ $$, and the other eigenvalues are the characteristic roots of $\ \left| {hE - J({E^*})} \right|$, where:

22 $$\begin{aligned} \begin{aligned} \ \left| {hE - J({E^*})} \right| = \left| {\begin{array}{*{20}{c}} {h + \alpha {\varphi ^*} + \beta {\varphi ^*} + \mu }&{}{\alpha {S^*} + \beta {S^*}}&{}{\alpha {S^*} + \beta {S^*}}\\ { - \alpha {\varphi ^*}}&{}{h - \alpha {S^*} - ({\gamma _{11}} + {\gamma _{12}} + \mu )}&{}{ - \alpha {S^*}}\\ { - \beta {\varphi ^*}}&{}{ - \beta {S^*}}&{}{h - \beta {S^*} - ({\gamma _{22}} + {\gamma _{21}} + \mu )} \end{array}} \right| .\ \end{aligned} \end{aligned}$$

The eigenvalues of Eq. (22) can be obviously obtained as:

23 $$\begin{aligned} {\left| {hE - J({E^*})} \right| }&= {{h^3} +\bigg [\mu + ({\gamma _{11}} + {\gamma _{12}} + \mu ) + ({\gamma _{22}} + {\gamma _{21}} + \mu ) + \alpha ({\varphi ^*} - {S^*})}\\ \nonumber&\quad + {\beta ({\varphi ^*} - {S^*})\bigg ]{h^2} + \bigg \{ \mu ({\gamma _{11}} + {\gamma _{12}} + \mu + {\gamma _{22}} + {\gamma _{21}} + \mu )}\\ \nonumber&\quad + {({\gamma _{11}} + {\gamma _{12}} + \mu )({\gamma _{22}} + {\gamma _{21}} + \mu ) + \alpha ({\gamma _{22}} + {\gamma _{21}} + \mu )({\varphi ^*} - {S^*})}\\\nonumber&\quad +{\beta ({\gamma _{11}} + {\gamma _{12}} + \mu )({\varphi ^*} - {S^*}) + \alpha \bigg [({\gamma _{11}} + {\gamma _{12}} + \mu ){\varphi ^*} - \mu {S^*}\bigg ]}\\\nonumber&\quad + {\beta \bigg [({\gamma _{22}} + {\gamma _{21}} + \mu ){\varphi ^*} - \mu {S^*}\bigg ]\bigg \} h + \bigg \{ \mu ({\gamma _{11}} + {\gamma _{12}} + \mu )({\gamma _{22}}}\\\nonumber&\quad + {{\gamma _{21}} + \mu ) + \alpha ({\gamma _{22}} + {\gamma _{21}} + \mu )\bigg [({\gamma _{11}} + {\gamma _{12}} + \mu ){\varphi ^*} - \mu {S^*}\bigg ]}\\\nonumber&\quad + {\beta ({\gamma _{11}} + {\gamma _{12}} + \mu )\bigg [({\gamma _{22}} + {\gamma _{21}} + \mu ){\varphi ^*} - \mu {S^*}\bigg ]\bigg \}.} \end{aligned}$$

Then we construct a cubic polynomial and replace the coefficient with ${a_3},{a_2},{a_1},{a_0}$ to determine the other eigenvalues of System (21). Hence, Eq. (23) can be rewritten as:

24 $$\begin{aligned} \begin{aligned} \ {a_3}{h^3} + {a_2}{h^2} + {a_1}h + {a_0} = 0,\ \end{aligned} \end{aligned}$$

where:

25 $$\begin{aligned} \ {a_3}&= 1,\ \end{aligned}$$

26 $$\begin{aligned} \ {a_2}&= [\mu + ({\gamma _{11}} + {\gamma _{12}} + \mu ) + ({\gamma _{22}} + {\gamma _{21}} + \mu ) + \alpha ({\varphi ^*} - {S^*}) + \beta ({\varphi ^*} - {S^*})],\ \end{aligned}$$

27 $$\begin{aligned} {{a_1}}&= {\mu ({\gamma _{11}} + {\gamma _{12}} + \mu + {\gamma _{22}} + {\gamma _{21}} + \mu ) + ({\gamma _{11}} + {\gamma _{12}} + \mu )({\gamma _{22}} + {\gamma _{21}} + \mu )}\\ \nonumber {}&\quad + {\alpha ({\gamma _{22}} + {\gamma _{21}} + \mu )({\varphi ^*} - {S^*}) + \beta ({\gamma _{11}} + {\gamma _{12}} + \mu )({\varphi ^*} - {S^*})}\\ \nonumber {}&\quad + {\alpha [({\gamma _{11}} + {\gamma _{12}} + \mu ){\varphi ^*} - \mu {S^*}] + \beta [({\gamma _{22}} + {\gamma _{21}} + \mu ){\varphi ^*} - \mu {S^*}],} \end{aligned}$$

28 $$\begin{aligned} {{a_0}}&= {\mu ({\gamma _{11}} + {\gamma _{12}} + \mu )({\gamma _{22}} + {\gamma _{21}} + \mu ) + \alpha ({\gamma _{22}} + {\gamma _{21}} + \mu )[({\gamma _{11}} + {\gamma _{12}}}\\ \nonumber {}&\quad + {\mu ){\varphi ^*} - \mu {S^*}] + \beta ({\gamma _{11}} + {\gamma _{12}} + \mu )[({\gamma _{22}} + {\gamma _{21}} + \mu ){\varphi ^*} - \mu {S^*}]}, \end{aligned}$$

then, let

29 $$\begin{aligned} \ \left\{ {\begin{array}{*{20}{l}} {{\omega _1}}&{} = &{}{\mu + ({\gamma _{11}} + {\gamma _{12}} + \mu ) + ({\gamma _{22}} + {\gamma _{21}} + \mu ),}\\ {{\omega _2}}&{} = &{}{\alpha ({\varphi ^*} - {S^*}),}\\ {{\omega _3}}&{} = &{}{\beta ({\varphi ^*} - {S^*}),}\\ {{\omega _4}}&{} = &{}{\mu ({\gamma _{11}} + {\gamma _{12}} + \mu + {\gamma _{22}} + {\gamma _{21}} + \mu ) + ({\gamma _{11}} + {\gamma _{12}} + \mu )({\gamma _{22}} + {\gamma _{21}} + \mu ),}\\ {{\omega _5}}&{} = &{}{\alpha [({\gamma _{11}} + {\gamma _{12}} + \mu ){\varphi ^*} - \mu {S^*}],}\\ {{\omega _6}}&{} = &{}{\beta [({\gamma _{22}} + {\gamma _{21}} + \mu ){\varphi ^*} - \mu {S^*}],} \end{array}} \right. \ \end{aligned} $$

and

30 $$\begin{aligned} {{a_2}{a_1} - {a_3}{a_0}}&={{\omega _1}{\omega _4} + ({\gamma _{22}} + {\gamma _{21}} + \mu ){\omega _1}{\omega _2} + ({\gamma _{11}} + {\gamma _{12}} + \mu ){\omega _1}{\omega _3}}\\ \nonumber {}&\quad +{{\omega _2}{\omega _4} + ({\gamma _{22}} + {\gamma _{21}} + \mu )\omega _2^2 + ({\gamma _{11}} + {\gamma _{12}} + \mu ){\omega _2}{\omega _3}}\\ \nonumber {}&\quad + {{\omega _3}{\omega _4} + ({\gamma _{22}} + {\gamma _{21}} + \mu ){\omega _2}{\omega _3} + ({\gamma _{11}} + {\gamma _{12}} + \mu )\omega _3^2}\\ \nonumber {}&\quad - {\mu ({\gamma _{11}} + {\gamma _{12}} + \mu )({\gamma _{22}} + {\gamma _{21}} + \mu ) + [{\omega _1} + {\omega _2} + {\omega _3}}\\ \nonumber {}&\quad - {({\gamma _{22}} + {\gamma _{21}} + \mu )]{\omega _5} + [{\omega _1} + {\omega _2} + {\omega _3} - ({\gamma _{11}} + {\gamma _{12}} + \mu )]{\omega _6}.} \end{aligned}$$

The condition of information-existence equilibrium point ${E^*} = ({S^*},I_1^*,I_2^*,M_1^*,M_2^*,M_3^*,M_4^*,{R^*})$ is locally asymptotically stable and the conditions: (i) ${a_3},{a_2},{a_1},{a_0} > 0$ and (ii) ${a_2}{a_1} - {a_3}{a_0} > 0$ based on the Routh–Hurwitz criterion. It is easy to know that ${a_3} > 0$.

If ${\mu ^2}{R_0}({R_0}-1) > B(\alpha + \beta )$ and ${R_0} > 1$, then ${a_2},{a_1},{a_0} > 0$ and ${a_2}{a_1} - {a_3}{a_0} > 0$. In this case, the Routh–Hurwitz criterion are satisfied. Hence, the information-existence equilibrium point ${E^*} = ({S^*},I_1^*,I_2^*,M_1^*,M_2^*,M_3^*,M_4^*,{R^*})$ of System (1) is locally asymptotically stable. $\square$

### Theorem 4

If $R_0>1$, the information-existence equilibrium point ${E^*} = ({S^*},I_1^*,I_2^*,M_1^*,M_2^*,M_3^*,M_4^*,{R^*})$ of System (1) is globally asymptotically stable.

### Proof 4

We construct the Lyapunov function as:

31 $$\begin{aligned} {W(t)}&= {[(S(t) - {S^*}) + ({I_1}(t) - I_1^*) + ({I_2}(t) - I_2^*) + ({M_1}(t) - M_1^*)}\\ \nonumber {}&\quad + {({M_2}(t) - M_2^*) + ({M_3}(t) - M_3^*) + ({M_4}(t) - M_4^*) + (R(t) - {R^*}){]^2}}, \end{aligned}$$

and

32 $$\begin{aligned} {W'(t)}&={2[(S(t) - {S^*}) + ({I_1}(t) - I_1^*) + ({I_2}(t) - I_2^*) + ({M_1}(t) - M_1^*)}\\\nonumber {}&\quad + {({M_2}(t) - M_2^*) + ({M_3}(t) - M_3^*) + ({M_4}(t) - M_4^*) + (R(t)}\\\nonumber {}&\quad - {{R^*})][S'(t) + {I_1}'(t) + {I_2}'(t) + {M_1}'(t) + {M_2}'(t) + {M_3}'(t) + {M_4}'(t) + R(t)]}\\\nonumber {}&={2[(S(t) - {S^*}) + ({I_1}(t) - I_1^*) + ({I_2}(t) - I_2^*) + ({M_1}(t) - M_1^*)}\\\nonumber {}&\quad + {({M_2}(t) - M_2^*) + ({M_3}(t) - M_3^*) + ({M_4}(t) - M_4^*) + (R(t)}\\\nonumber {}&\quad - {{R^*})][B - \mu S - \mu {I_1} - \mu {I_2} - \mu {M_1} - \mu {M_2} - \mu {M_3} - \mu {M_4} - \mu R].}\\\nonumber \end{aligned}$$

Because of the existence of ${E^*} = ({S^*},I_1^*,I_2^*,M_1^*,M_2^*,M_3^*,M_4^*,{R^*})$, we can know that $B - \mu {S^\mathrm{{*}}} - \mu I_1^\mathrm{{*}} - \mu I_2^\mathrm{{*}} - \mu M_1^\mathrm{{*}} - \mu M_2^\mathrm{{*}} - \mu M_3^\mathrm{{*}} - \mu M_4^\mathrm{{*}} - \mu {R^\mathrm{{*}}}\mathrm{{ = }}0$, i.e., $B\mathrm{{ = }}\mu {S^\mathrm{{*}}} + \mu I_1^\mathrm{{*}} + \mu I_2^\mathrm{{*}} + \mu M_1^\mathrm{{*}} + \mu M_2^\mathrm{{*}} + \mu M_3^\mathrm{{*}} + \mu M_4^\mathrm{{*}} + \mu {R^\mathrm{{*}}}$.

Then, Eq. (32) can be computed as:

33 $$\begin{aligned} {W'(t)}&= {2[(S(t) - {S^*}) + ({I_1}(t) - I_1^*) + ({I_2}(t) - I_2^*) + ({M_1}(t) - M_1^*)}\\\nonumber {}&\quad + {({M_2}(t) - M_2^*) + ({M_3}(t) - M_3^*) + ({M_4}(t) - M_4^*) + (R(t)}\\\nonumber {}&\quad - {{R^*})][\mu {S^*} + \mu I_1^* + \mu I_2^* + \mu M_1^* + \mu M_2^* + \mu M_3^* + \mu M_4^* + \mu {R^*}}\\\nonumber {}&\quad - {\mu S - \mu {I_1} - \mu {I_2} - \mu {M_1} - \mu {M_2} - \mu {M_3} - \mu {M_4} - \mu R]}\\\nonumber {}&= { - 2[(S - {S^*}) + ({I_1} - I_1^*) + ({I_2} - I_2^*) + ({M_1} - M_1^*)}\\\nonumber {}&\quad + {({M_2} - M_2^*) + ({M_3} - M_3^*) + ({M_4} - M_4^*) + {{(R - {R^*})}^2}.} \end{aligned}$$

Besides that, $W'(t) = 0$ holds if and only if $S(t) = {S^*},{I_1}(t) = I_1^*,{I_2}(t) = I_2^*,{M_1}(t) = M_1^*,{M_2}(t) = M_2^*,{M_3}(t) = M_3^*,{M_4}(t) = M_4^*,R(t) = {R^*}$. Hence, the information-existence equilibrium point ${E^*} = ({S^*},I_1^*,I_2^*,M_1^*,M_2^*,M_3^*,M_4^*,{R^*})$ of System (1) is globally asymptotically stable based on Lyapunov-LaSalle Invariance Principle^30^. $\square$

## The optimal control model

Based on the information transmission model established above, we consider the fact that the educational fields all over the world encourage interdisciplinary application. This is consistent with the information crossing we have constructed and the new variant information that has been generated. Therefore, in order to promote large-scale information crossing and strengthen the generation of variant information after crossing, two control objectives are accordingly proposed. On one hand, there are more and more people who are exposed to cross information, and on the other hand, there are more and more variations that fuse the two kinds of information. For this reason, the four proportionality constants $\alpha$, $\beta$, $\gamma _{12}$, and $\gamma _{21}$ in the model are transformed into control variables $\alpha (t)$, $\beta (t)$, $\gamma _{12}(t)$, and $\gamma _{21}(t)$, respectively. The control variable $\alpha (t)$ is used to control the rate of exposure to the first kind of information and then to the second kind of information. Similarly, the control variable $\beta (t)$ is used to control the rate of exposure to the second kind of information and then to the first kind of information. Generally, the rate of exposure is improved by improving the flow of people or organizing the information exchange activities. The control variable $\gamma _{12}(t)$ is used to control the rate of variation that considers the first kind of information as the main and the second information as the auxiliary. Moreover, the control variable $\gamma _{21}(t)$ is used to control the rate of variation that considers the second kind of information as the main and the first kind of information as the auxiliary. Generally, the variation rate can be improved based on educational guidance or policy encouragement.

Hence, an objective function can be proposed as:

34 $$\begin{aligned} \begin{aligned} \ \begin{array}{*{20}{l}} {J(\alpha ,\beta ,{\gamma _{12}},{\gamma _{21}})}&{} = &{}{\int _0^{{t_f}} {[{I_1}(t) + {I_2}(t) + {M_2}(t) + {M_3}(t) - {{{{c_1}}} \!{/}}\!{2}{\alpha ^2}(t)} }\\ {}&{} - &{}{{{{{c_2}}} \!{/}\!{2}}{\beta ^2}(t) - {{{{c_3}}} \!{/} \!{2}}\gamma _{12}^2(t) - {{{{c_4}}} \!{/} \!{2}}\gamma _{21}^2(t)],} \end{array} \end{aligned} \end{aligned}$$

and satisfy the follow state system

35 $$\begin{aligned} \begin{aligned} \ \left\{ {\begin{array}{*{20}{l}} {\frac{{dS}}{{dt}}}&{} = &{}{B - \alpha (t) ({I_1} + {I_2})S - \beta (t) ({I_1} + {I_2})S - \mu S},\\ {\frac{{d{I_1}}}{{dt}}}&{} = &{}{\alpha (t) ({I_1} + {I_2})S - {\gamma _{11}}{I_1} - {\gamma _{12}}(t){I_1} - \mu {I_1}},\\ {\frac{{d{I_2}}}{{dt}}}&{} = &{}{\beta (t) ({I_1} + {I_2})S - {\gamma _{22}}{I_2} - {\gamma _{21}}(t){I_2} - \mu {I_2}},\\ {\frac{{d{M_1}}}{{dt}}}&{} = &{}{{\gamma _{11}}{I_1} - {\varepsilon _1}{M_1} - \mu {M_1}},\\ {\frac{{d{M_2}}}{{dt}}}&{} = &{}{{\gamma _{12}}(t){I_1} - {\varepsilon _2}{M_2} - \mu {M_2}},\\ {\frac{{d{M_3}}}{{dt}}}&{} = &{}{{\gamma _{21}}(t){I_2} - {\varepsilon _3}{M_3} - \mu {M_3}},\\ {\frac{{d{M_4}}}{{dt}}}&{} = &{}{{\gamma _{22}}{I_2} - {\varepsilon _4}{M_4} - \mu {M_4}},\\ {\frac{{dR}}{{dt}}}&{} = &{}{{\varepsilon _1}{M_1} + {\varepsilon _2}{M_2} + {\varepsilon _3}{M_3} + {\varepsilon _4}{M_4} - \mu R.} \end{array}} \right. \ \end{aligned} \end{aligned}$$

The initial conditions for System (35) are satisfied:

36 $$\begin{aligned} \begin{aligned} \ \begin{array}{*{20}{l}} {S(0) = {S_0},{I_1}(0) = {I_{1,0}},{I_2}(0) = {I_{2,0}},{M_1}(0) = {M_{1,0}},{M_2}(0) = {M_{2,0}},}\\ {{M_3}(0) = {M_{3,0}},{M_4}(0) = {M_{4,0}},R(0) = {R_0},} \end{array}\ \end{aligned} \end{aligned}$$

where:

37 $$\begin{aligned} \begin{aligned} \ \begin{array}{*{20}{l}} {\alpha (t),\beta (t),{\gamma _{12}}(t),{\gamma _{21}}(t)}&{} \in &{}{U \buildrel \Delta \over = \{ (\alpha ,\beta ,{\gamma _{12}},{\gamma _{21}})\vert (\alpha (t),\beta (t),{\gamma _{12}}(t),{\gamma _{21}}(t))}\\ {}&{}{}&{}{measurable,0 \le \alpha (t),\beta (t),{\gamma _{12}}(t),{\gamma _{21}}(t) \le 1,}\\ {}&{}{}&{}{\forall t \in [0,{t_f}]\},} \end{array}\ \end{aligned} \end{aligned}$$

while *U* is the admissible control set. The time interval of control is between 0 and $t_f$. $c_1$, $c_2$, $c_3$, $c_4$ are positive weight coefficients shown the control strength and importance of four control measures.

### Theorem 5

An optimal control pair $({\alpha ^*},{\beta ^*},{{\gamma _{12}} ^*},{{\gamma _{21}} ^*}) \in U$ exists so that the function is established below:

38 $$\begin{aligned} \begin{aligned} \ J({\alpha ^*},{\beta ^*},\gamma _{12}^*,\gamma _{21}^*) = \max \{ J(\alpha ,\beta ,{\gamma _{12}},{\gamma _{21}}):(\alpha ,\beta ,{\gamma _{12}},{\gamma _{21}}) \in U\}.\ \end{aligned} \end{aligned}$$

### Proof 5

Let $$X(t) = {(S(t),{I_1}(t),{I_2}(t),{M_1}(t),{M_2}(t),{M_3}(t),{M_4}(t),R(t))^T}$$ and

39 $$\begin{aligned} \begin{aligned} \ \begin{array}{*{20}{l}} {L(t;X(t),\alpha (t),\beta (t),{\gamma _{12}}(t),{\gamma _{21}}(t))}&{} = &{}{{I_1}(t) + {I_2}(t) + {M_2}(t) + {M_3}(t) - {{{{c_1}}} \!{/}\!{2}}{\alpha ^2}(t)}\\ {}&{} - &{}{{{{{c_2}}} \!{/}\!{2}}{\beta ^2}(t) - {{{{c_3}}} \!{/}\!{2}}\gamma _{12}^2(t) - {{{{c_4}}} \!{/} \!{2}}\gamma _{21}^2(t).} \end{array}\ \end{aligned} \end{aligned}$$

The existence of an optimal pair must satisfy: (i) the set of control variables and state variables is nonempty, (ii) the control set *U* is convex and closed, (iii) the right-hand side of the state system is bounded by a linear function in the state and control variables, (iv) the integrand of the objective functional is convex on *U*, (v) there exist constants ${d_1},{d_2} > 0$ and $\rho > 1$ such that the integrand of the objective functional satisfies:

40 $$\begin{aligned} \begin{aligned} \ - L(t;X(t),\alpha ;\beta ;{\gamma _{12}};{\gamma _{21}}) \ge {d_1}{({\left| \alpha \right| ^2} + {\left| \beta \right| ^2} + {\left| {{\gamma _{12}}} \right| ^2} + {\left| {{\gamma _{21}}} \right| ^2})^ {{\rho } \!{/}\!{2}}} - {d_2}.\ \end{aligned} \end{aligned}$$

Conditions (i)-(iii) are clearly established, we just prove the condition (iv) and (v). One can easily obtain inequality:

41 $$\begin{aligned} \begin{aligned} \ \begin{array}{*{20}{l}} {S' \le B,{I_1}^\prime \le \alpha (t)({I_1} + {I_2})S,{I_2}^\prime \le \beta (t)({I_1} + {I_2})S,{M_1}^\prime \le {\gamma _{11}}{I_1},{M_2}^\prime \le {\gamma _{12}}(t){I_1},}\\ {{M_3}^\prime \le {\gamma _{21}}(t){I_2},{M_4}^\prime \le {\gamma _{22}}{I_1},R' \le {\varepsilon _1}{M_1} + {\varepsilon _2}{M_2} + {\varepsilon _3}{M_3} + {\varepsilon _4}{M_4}.} \end{array}\ \end{aligned} \end{aligned}$$

Hence, condition (iv) is established. Then, for any $t \ge 0$, there is a positive constant *M* which is satisfied $\left| {X(t)} \right| \le M$, therefore

42 $$\begin{aligned} \begin{aligned} { - L(t;X(t),\alpha ;\beta ;{\gamma _{12}};{\gamma _{21}})} =&\, {{ \!{{{{({c_1}{\alpha ^2}(t) + {c_2}{\beta ^2}(t) + {c_3}\gamma _{12}^2(t) + {c_4}\gamma _{21}^2(t))} /2}}} \!}}\\ {}&- {{I_1}(t) - {I_2}(t) - {M_2}(t) - {M_3}(t)}\\ {} \ge&\,{{d_1}{{({{\left| \alpha \right| }^2} + {{\left| \beta \right| }^2} + {{\left| {{\gamma _{12}}} \right| }^2} + {{\left| {{\gamma _{21}}} \right| }^2})}^ {{{\rho } \!{/}\!{2}}}} - 2M.} \end{aligned} \end{aligned}$$

Let ${d_1} = \min \left\{ {\frac{{{c_1}}}{2},\frac{{{c_2}}}{2},\frac{{{c_3}}}{2},\frac{{{c_4}}}{2}} \right\} ,{d_2} = 2M$ and $\rho = 2$, then condition (v) is established. Hence, the optimal control can be realized. $\square$

### Theorem 6

For the optimal control pair $({\alpha ^*},{\beta ^*},{\gamma _{12} ^*},{\gamma _{21} ^*})$ of state System (35), there exist adjoint variables ${\delta _1},{\delta _2},{\delta _3},{\delta _4},{\delta _5},{\delta _6},$

$${\delta _7},{\delta _8}$$ that satisfy:

43 $$\begin{aligned} \begin{aligned} \ \left\{ {\begin{array}{*{20}{l}} {\frac{{d{\delta _1}}}{{dt}}}&{} = &{}{({\delta _1} - {\delta _2})\alpha (t)({I_1} + {I_2}) + ({\delta _1} - {\delta _3})\beta (t)({I_1} + {I_2}) + {\delta _1}\mu ,}\\ {\frac{{d{\delta _2}}}{{dt}}}&{} = &{}{1 + ({\delta _1} - {\delta _2})\alpha (t)S + ({\delta _1} - {\delta _3})\beta (t)S + ({\delta _2} - {\delta _4}){\gamma _{11}} + ({\delta _2} - {\delta _5}){\gamma _{12}}(t) + {\delta _2}\mu ,}\\ {\frac{{d{\delta _3}}}{{dt}}}&{} = &{}{1 + ({\delta _1} - {\delta _2})\alpha (t)S + ({\delta _1} - {\delta _3})\beta (t)S + ({\delta _3} - {\delta _7}){\gamma _{22}} + ({\delta _3} - {\delta _6}){\gamma _{21}}(t) + {\delta _3}\mu ,}\\ {\frac{{d{\delta _4}}}{{dt}}}&{} = &{}{({\delta _4} - {\delta _8}){\varepsilon _1} + {\varepsilon _4}\mu ,}\\ {\frac{{d{\delta _5}}}{{dt}}}&{} = &{}{1 + ({\delta _5} - {\delta _8}){\varepsilon _2} + {\varepsilon _5}\mu ,}\\ {\frac{{d{\delta _6}}}{{dt}}}&{} = &{}{1 + ({\delta _6} - {\delta _8}){\varepsilon _3} + {\varepsilon _6}\mu ,}\\ {\frac{{d{\delta _7}}}{{dt}}}&{} = &{}{({\delta _7} - {\delta _8}){\varepsilon _4} + {\varepsilon _7}\mu ,}\\ {\frac{{d{\delta _8}}}{{dt}}}&{} = &{}{{\delta _8}\mu .} \end{array}} \right. \ \end{aligned} \end{aligned}$$

With boundary conditions:

44 $$\begin{aligned} \begin{aligned} \ {\delta _1}({t_f}) = {\delta _2}({t_f}) = {\delta _3}({t_f}) = {\delta _4}({t_f}) = {\delta _5}({t_f})= {\delta _6}({t_f})= {\delta _7}({t_f})= {\delta _8}({t_f}) = 0.\ \end{aligned} \end{aligned}$$

In addition, the optimal control pair $({\alpha ^*},{\beta ^*},{\gamma _{12} ^*},{\gamma _{21} ^*})$ of state System (35) can be given by:

45 $$\begin{aligned} & \begin{aligned} \ {\alpha ^*}(t) = \min \left\{ {1,\max \left\{ {0,\frac{{({\delta _1} - {\delta _2})({I_1} + {I_2})S}}{{{c_1}}}} \right\} } \right\} ,\ \end{aligned} \end{aligned}$$

46 $$\begin{aligned} & \begin{aligned} \ {\beta ^*}(t) = \min \left\{ {1,\max \left\{ {0,\frac{{({\delta _1} - {\delta _3})({I_1} + {I_2})S}}{{{c_2}}}} \right\} } \right\} ,\ \end{aligned} \end{aligned}$$

47 $$\begin{aligned} & \begin{aligned} \ \gamma _{12}^*(t) = \min \left\{ {1,\max \left\{ {0,\frac{{({\delta _2} - {\delta _5}){I_1}}}{{{c_3}}}} \right\} } \right\} ,\ \end{aligned} \end{aligned}$$

48 $$\begin{aligned} & \begin{aligned} \ \gamma _{21}^*(t) = \min \left\{ {1,\max \left\{ {0,\frac{{({\delta _3} - {\delta _6}){I_2}}}{{{c_4}}}} \right\} } \right\} .\ \end{aligned} \end{aligned}$$

### Proof 6

Define a Hamiltonian function enlarged with penalty term to obtain the expression of optimal control system and optimal control pair. The Hamiltonian function enlarged can be written as:

49 $$\begin{aligned} \begin{aligned} H =&\,{ - {I_1}(t) - {I_2}(t) - {M_2}(t) - {M_3}(t) + {{{{c_1}}} \!{/}\!{2}}{\alpha ^2}(t) + {{{{c_2}}} \!{/}\!{2}}{\beta ^2}(t) + {{{{c_3}}} \!{/}\!{2}}\gamma _{12}^2(t) + {{{{c_4}}} \!{/}\!{2}}\gamma _{21}^2(t)}\\ {}&+ \,{{\delta _1}[B - \alpha (t)({I_1} + {I_2})S - \beta (t)({I_1} + {I_2})S - \mu S] + {\delta _2}[\alpha (t)({I_1} + {I_2})S - {\gamma _{11}}{I_1}}\\ {}&- \,{{\gamma _{12}}(t){I_1} - \mu {I_1}] + {\delta _3}[\beta (t)({I_1} + {I_2})S - {\gamma _{22}}{I_2} - {\gamma _{21}}(t){I_2} - \mu {I_2}] + {\delta _4}[{\gamma _{11}}{I_1} - {\varepsilon _1}{M_1}}\\ {}&-\, {\mu {M_1}] + {\delta _5}[{\gamma _{12}}(t){I_1} - {\varepsilon _2}{M_2} - \mu {M_2}] + {\delta _6}[{\gamma _{21}}(t){I_2} - {\varepsilon _3}{M_3} - \mu {M_3}] + {\delta _7}[{\gamma _{22}}{I_1}}\\ {}&-\, {{\varepsilon _4}{M_4} - \mu {M_4}] + {\delta _8}[{\varepsilon _1}{M_1} + {\varepsilon _2}{M_2} + {\varepsilon _3}{M_3} + {\varepsilon _4}{M_4} \!-\! \mu R] \!-\! {\lambda _{11}}\alpha (t) \!-\! {\lambda _{12}}(1 \!-\! \alpha (t))}\\ {}&- \,{{\lambda _{21}}\beta (t) - {\lambda _{22}}(1 - \beta (t)) - {\lambda _{31}}{\gamma _{12}}(t) \!-\! {\lambda _{32}}(1 \!-\! {\gamma _{12}}(t)) \!-\! {\lambda _{41}}{\gamma _{21}}(t) \!-\! {\lambda _{42}}(1 \!-\! {\gamma _{21}}(t)),} \end{aligned} \end{aligned}$$

which the penalty term is ${\lambda _{ij}}(t) \ge 0$ , and it is satisfied that ${\lambda _{11}}(t){\alpha }(t) = {\lambda _{12}}(t)(1 - {\alpha }(t)) = 0$ at optimal control ${\alpha ^*}$, ${\lambda _{21}}(t)\beta (t) = {\lambda _{22}}(t)(1 - \beta (t)) = 0$ at optimal control ${\beta ^*}$, ${\lambda _{31}}(t)\gamma _{12} (t) = {\lambda _{32}}(t)(1 - \gamma _{12} (t)) = 0$ at optimal control ${\gamma _{12} ^*}$ and ${\lambda _{41}}(t)\gamma _{21} (t) = {\lambda _{42}}(t)(1 - \gamma _{21} (t)) = 0$ at optimal control ${\gamma _{21} ^*}$.

Based on the Pontyragin maximum principle, the adjoint system can be written as:

50 $$\begin{aligned} \begin{aligned} \ \begin{array}{*{20}{l}} {\frac{{d{\delta _1}}}{{dt}} = - \frac{{\partial H}}{{\partial S}},\frac{{d{\delta _2}}}{{dt}} = - \frac{{\partial H}}{{\partial {I_1}}},\frac{{d{\delta _3}}}{{dt}} = - \frac{{\partial H}}{{\partial {I_2}}},\frac{{d{\delta _4}}}{{dt}} = - \frac{{\partial H}}{{\partial {M_1}}},}\\ {\frac{{d{\delta _5}}}{{dt}} = - \frac{{\partial H}}{{\partial {M_2}}},\frac{{d{\delta _6}}}{{dt}} = - \frac{{\partial H}}{{\partial {M_3}}},\frac{{d{\delta _7}}}{{dt}} = - \frac{{\partial H}}{{\partial {M_4}}},\frac{{d{\delta _8}}}{{dt}} = - \frac{{\partial H}}{{\partial R}},} \end{array}\ \end{aligned} \end{aligned}$$

and the boundary conditions of adjoint system are

51 $$\begin{aligned} \begin{aligned} \ {\delta _1}({t_f}) = {\delta _2}({t_f}) = {\delta _3}({t_f}) = {\delta _4}({t_f}) = {\delta _5}({t_f})= {\delta _6}({t_f})= {\delta _7}({t_f})= {\delta _8}({t_f}) = 0.\ \end{aligned} \end{aligned}$$

Let ${\alpha ^*}$ as an example to give the optimality conditions. One have

52 $$\begin{aligned} \begin{aligned} \ \frac{{\partial H}}{{\partial \alpha }} = {c_1}\alpha (t) + {\delta _1}[ - ({I_1} + {I_2})S] + {\delta _2}[({I_1} + {I_2})S] - {\lambda _{11}} + {\lambda _{12}} = 0,\ \end{aligned} \end{aligned}$$

and the optimal control formulae can be written as:

53 $$\begin{aligned} \begin{aligned} \ {\alpha ^*} = \frac{1}{{{c_1}}}({\delta _1} - {\delta _2})({I_1} + {I_2})S + {\lambda _{11}} - {\lambda _{12}}.\ \end{aligned} \end{aligned}$$

To obtain the final optimal control formulae without ${\lambda _{11}}$ and ${\lambda _{12}}$ need to consider the following three situations.

The first situation is that ${\lambda _{11}}(t) = {\lambda _{12}}(t) = 0$ in set $\left\{ {\left. t \right| 0< {\alpha ^*}(t) < 1} \right\}$, then the optimal control formulae can be written as:

54 $$\begin{aligned} \begin{aligned} \ {\alpha ^*}(t) = \frac{1}{{{c_1}}}({\delta _1} - {\delta _2})({I_1} + {I_2})S.\ \end{aligned} \end{aligned}$$

The second situation is that ${\lambda _{11}}(t) = 0$ in set $\left\{ {\left. t \right| {\alpha ^*}(t) = 1} \right\}$, then the optimal control formulae can be written as:

55 $$\begin{aligned} \begin{aligned} \ 1 = {\alpha ^*}(t) = \frac{1}{{{c_1}}}[({\delta _1} - {\delta _2})({I_1} + {I_2})S - {\lambda _{12}}].\ \end{aligned} \end{aligned}$$

Due to ${\lambda _{12}}(t) \ge 0$, it is shown that $\frac{1}{{{c_1}}}({\delta _1} - {\delta _2})({I_1} + {I_2})S \ge 1$.

The third situation is that ${\lambda _{12}}(t) = 0$ in set $\left\{ {\left. t \right| {\alpha ^*}(t) = 0} \right\}$, then the optimal control formulae can be written as:

56 $$\begin{aligned} \begin{aligned} \ 0 = {\alpha ^*}(t) = \frac{1}{{{c_1}}}[({\delta _1} - {\delta _2})({I_1} + {I_2})S + {\lambda _{11}}].\ \end{aligned} \end{aligned}$$

Based on the above situation, the final optimal control formulae of ${\alpha ^*}(t)$ can be written as

${\alpha ^*}(t) = \min \left\{ {1,\max \left\{ {0,\frac{{({\delta _1} - {\delta _2})({I_1} + {I_2})S}}{{{c_1}}}} \right\} } \right\}$. Similarly, the final optimal control formulae of ${\beta ^*}(t)$ can be written as

${\beta ^*}(t) = \min \left\{ {1,\max \left\{ {0,\frac{{({\delta _1} - {\delta _3})({I_1} + {I_2})S}}{{{c_2}}}} \right\} } \right\}$, the final optimal control formulae of ${\gamma _{12} ^*}(t)$ can be written as

$\gamma _{12}^*(t) = \min \left\{ {1,\max \left\{ {0,\frac{{({\delta _2} - {\delta _5}){I_1}}}{{{c_3}}}} \right\} } \right\}$, the final optimal control formulae of ${\gamma _{21} ^*}(t)$ can be written as

$$\gamma _{21}^*(t) = \min \left\{ {1,\max \left\{ {0,\frac{{({\delta _3} - {\delta _6}){I_2}}}{{{c_4}}}} \right\} } \right\} $$.

So far, we get the optimal control system includes state System (35) with the initial conditions $S(0),{I_1}(0),{I_2}(0),{M_1}(0),{M_2}(0),{M_3}(0),{M_4}(0),R(0)$ and the adjoint System (43) with boundary conditions with the optimization conditions. The optimal control system can be written as:

57 $$ \begin{aligned} \ \left\{ {\begin{array}{*{20}{l}} {\frac{{dS}}{{dt}}}&{} = &{}{B - \min \left\{ {1,\max \left\{ {0,\frac{{({\delta _1} - {\delta _2})({I_1} + {I_2})S}}{{{c_1}}}} \right\} } \right\} (t)({I_1} + {I_2})S}\\ {}&\quad{} - &{}{\min \left\{ {1,\max \left\{ {0,\frac{{({\delta _1} - {\delta _3})({I_1} + {I_2})S}}{{{c_2}}}} \right\} } \right\} (t)({I_1} + {I_2})S - \mu S,}\\ {\frac{{d{I_1}}}{{dt}}}&{} = &{}{\min \left\{ {1,\max \left\{ {0,\frac{{({\delta _1} - {\delta _2})({I_1} + {I_2})S}}{{{c_1}}}} \right\} } \right\} (t)({I_1} + {I_2})S - {\gamma _{11}}{I_1}}\\ {}&\quad{} - &{}{\min \left\{ {1,\max \left\{ {0,\frac{{({\delta _2} - {\delta _5}){I_1}}}{{{c_3}}}} \right\} } \right\} (t){I_1} - \mu {I_1},}\\ {\frac{{d{I_2}}}{{dt}}}&{} = &{}{\min \left\{ {1,\max \left\{ {0,\frac{{({\delta _1} - {\delta _3})({I_1} + {I_2})S}}{{{c_2}}}} \right\} } \right\} (t)({I_1} + {I_2})S - {\gamma _{22}}{I_2}}\\ {}&\quad{} - &{}{\min \left\{ {1,\max \left\{ {0,\frac{{({\delta _3} - {\delta _6}){I_2}}}{{{c_4}}}} \right\} } \right\} (t){I_2} - \mu {I_2},}\\ {\frac{{d{M_1}}}{{dt}}}&{} = &{}{{\gamma _{11}}{I_1} - {\varepsilon _1}{M_1} - \mu {M_1},}\\ {\frac{{d{M_2}}}{{dt}}}&{} = &{}{\min \left\{ {1,\max \left\{ {0,\frac{{({\delta _2} - {\delta _5}){I_1}}}{{{c_3}}}} \right\} } \right\} (t){I_1} - {\varepsilon _2}{M_2} - \mu {M_2},}\\ {\frac{{d{M_3}}}{{dt}}}&{} = &{}{\min \left\{ {1,\max \left\{ {0,\frac{{({\delta _3} - {\delta _6}){I_2}}}{{{c_4}}}} \right\} } \right\} (t){I_2} - {\varepsilon _3}{M_3} - \mu {M_3},}\\ {\frac{{d{M_4}}}{{dt}}}&{} = &{}{{\gamma _{22}}{I_2} - {\varepsilon _4}{M_4} - \mu {M_4},}\\ {\frac{{dR}}{{dt}}}&{} = &{}{{\varepsilon _1}{M_1} + {\varepsilon _2}{M_2} + {\varepsilon _3}{M_3} + {\varepsilon _4}{M_4} - \mu R,}\\ {\frac{{d{\delta _1}}}{{dt}}}&{} = &{}{({\delta _1} - {\delta _2})\min \left\{ {1,\max \left\{ {0,\frac{{({\delta _1} - {\delta _2})({I_1} + {I_2})S}}{{{c_1}}}} \right\} } \right\} (t)({I_1} + {I_2})}\\ {}&\quad{} + &{}{({\delta _1} - {\delta _3})\min \left\{ {1,\max \left\{ {0,\frac{{({\delta _1} - {\delta _3})({I_1} + {I_2})S}}{{{c_2}}}} \right\} } \right\} (t)({I_1} + {I_2}) + {\delta _1}\mu ,}\\ {\frac{{d{\delta _2}}}{{dt}}}&{} = &{}{1 + ({\delta _1} - {\delta _2})\min \left\{ {1,\max \left\{ {0,\frac{{({\delta _1} - {\delta _2})({I_1} + {I_2})S}}{{{c_1}}}} \right\} } \right\} (t)S}\\ {}&\quad{} + &{}{({\delta _1} - {\delta _3})\min \left\{ {1,\max \left\{ {0,\frac{{({\delta _1} - {\delta _3})({I_1} + {I_2})S}}{{{c_2}}}} \right\} } \right\} (t)S}\\ {}&\quad{} + &{}{({\delta _2} - {\delta _4}){\gamma _{11}} + ({\delta _2} - {\delta _5})\min \left\{ {1,\max \left\{ {0,\frac{{({\delta _2} - {\delta _5}){I_1}}}{{{c_3}}}} \right\} } \right\} (t) + {\delta _2}\mu ,}\\ {\frac{{d{\delta _3}}}{{dt}}}&{} = &{}{1 + ({\delta _1} - {\delta _2})\min \left\{ {1,\max \left\{ {0,\frac{{({\delta _1} - {\delta _2})({I_1} + {I_2})S}}{{{c_1}}}} \right\} } \right\} (t)S}\\ {}&\quad{} + &{}{({\delta _1} - {\delta _3})\min \left\{ {1,\max \left\{ {0,\frac{{({\delta _1} - {\delta _3})({I_1} + {I_2})S}}{{{c_2}}}} \right\} } \right\} (t)S}\\ {}&\quad{} + &{}{({\delta _3} - {\delta _7}){\gamma _{22}} + ({\delta _3} - {\delta _6})\min \left\{ {1,\max \left\{ {0,\frac{{({\delta _3} - {\delta _6}){I_2}}}{{{c_4}}}} \right\} } \right\} (t) + {\delta _3}\mu ,}\\ {\frac{{d{\delta _4}}}{{dt}}}&{} = &{}{({\delta _4} - {\delta _8}){\varepsilon _1} + {\varepsilon _4}\mu ,}\\ {\frac{{d{\delta _5}}}{{dt}}}&{} = &{}{1 + ({\delta _5} - {\delta _8}){\varepsilon _2} + {\varepsilon _5}\mu ,}\\ {\frac{{d{\delta _6}}}{{dt}}}&{} = &{}{1 + ({\delta _6} - {\delta _8}){\varepsilon _3} + {\varepsilon _6}\mu ,}\\ {\frac{{d{\delta _7}}}{{dt}}}&{} = &{}{({\delta _7} - {\delta _8}){\varepsilon _4} + {\varepsilon _7}\mu ,}\\ {\frac{{d{\delta _8}}}{{dt}}}&{} = &{}{{\delta _8}\mu ,} \end{array}} \right. \ \end{aligned}$$

and

58 $$\begin{aligned} & \begin{aligned} \ \begin{array}{*{20}{l}} {S(0) = {S_0},{I_1}(0) = {I_{1,0}},{I_2}(0) = {I_{2,0}},{M_1}(0) = {M_{1,0}},{M_2}(0) = {M_{2,0}},}\\ {{M_3}(0) = {M_{3,0}},{M_4}(0) = {M_{4,0}},R(0) = {R_0},} \end{array}\ \end{aligned} \end{aligned}$$

59 $$\begin{aligned} & \begin{aligned} \ {\delta _1}({t_f}) = {\delta _2}({t_f}) = {\delta _3}({t_f}) = {\delta _4}({t_f}) = {\delta _5}({t_f})= {\delta _6}({t_f})= {\delta _7}({t_f})= {\delta _8}({t_f}) = 0.\ \end{aligned} \end{aligned}$$

$$\square $$

## Numerical simulations

In this section, the Rung-Kutta algorithm is adopted for performing numerical simulations to verify the rationality of the theoretical results by MATLAB R2017b. It is noteworthy that the value range of parameters is not clearly defined in previous studies. Therefore, in this work, we combine the value of the basic regeneration number $R_0$ and the stability condition for obtaining and presenting the parameter values of the model.

**Figure 2:**
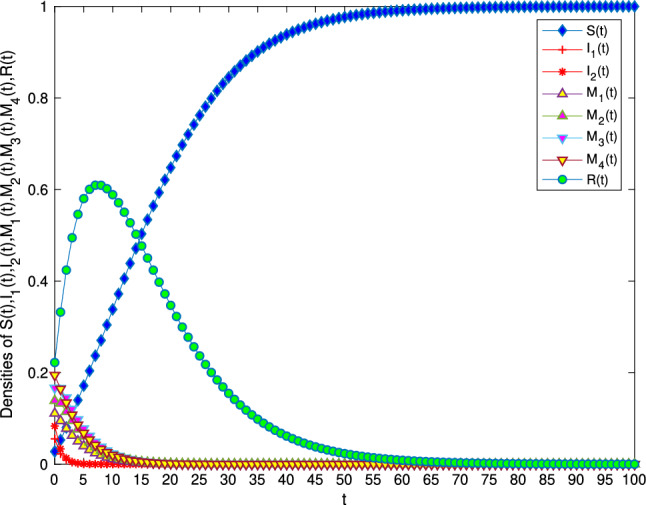
The stability of information-free equilibrium $E^0$ of system 1 with $R_0<1$.

In order to verify the locally and globally asymptotically stability of information-free equilibrium in Theorem 1 and Theorem 2. Let $B=1, \alpha =0.01, \beta =0.01, \mu =0.1, \gamma _{11}=0.2, \gamma _{12}=0.7, \gamma _{22}=0.2, \gamma _{21}=0.7, \varepsilon _1=\varepsilon _2=\varepsilon _3=\varepsilon _4=0.2$. It can be calculated that $R_0=0.2<1$. Figure 2 verifies the stability of the model and shows that variety groups eventually converge to 0 change over time.

**Figure 3:**
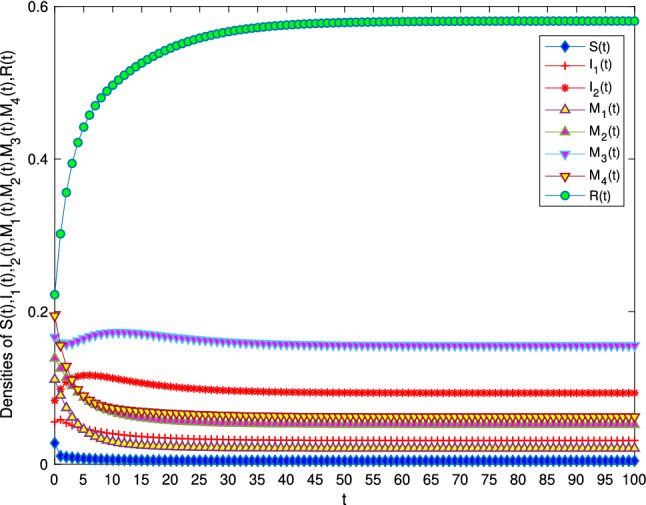
The stability of information-existence equilibrium $E^*$ of system 1 with $R_0>1$.

In order to verify the locally and globally asymptotically stability of information-existence equilibrium in Theorem 3 and Theorem 4. Let $B=3, \alpha =0.7, \beta =0.6, \mu =0.1, \gamma _{11}=0.2, \gamma _{12}=0.5, \gamma _{22}=0.2, \gamma _{21}=0.5, \varepsilon _1=\varepsilon _2=\varepsilon _3=\varepsilon _4=0.2$. It can be calculated that $R_0=48.75>1$. Figure 3 verifies the stability of the model and shows that variety groups eventually converge to $E^*$ change over time.

In order to analyze the effect of optimal control pair $(\alpha ^*,\beta ^*,\gamma _{12}^*,\gamma _{21}^*)$ on variety groups when adopt the optimal control strategy. One give the image of “optimal control $(\alpha =\alpha ^*(t),\beta =\beta ^*(t),\gamma _{12}^*(t),\gamma _{21}^*(t))$”, “middle control measure”, “single control measure” and “constant control measure” respectively.

**Figure 4:**
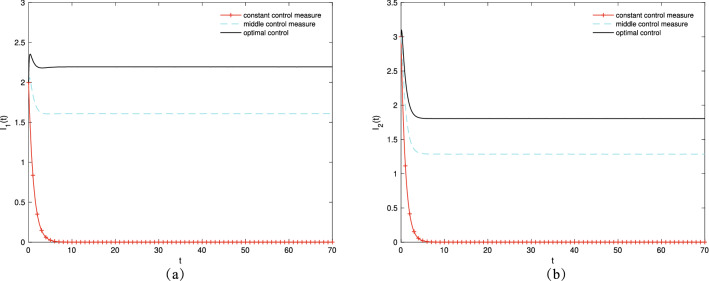
The densities of (**a**) $I_1(t)$, (**b**) $I_2(t)$ change over time under different control strategies.

First, different control strategies are adopted to increase the number of transmission groups $I_1$ and $I_2$. $\alpha ^*$ and $\beta ^*$ are controlled, respectively. Then, let $\beta =0.55, \mu =0.07, \gamma _{11}=0.2, \gamma _{12}=0.6, \gamma _{22}=0.2, \gamma _{21}=0.7, \varepsilon _1=0.16, \varepsilon _2=0.22, \varepsilon _3=0.18, \varepsilon _4=0.23$ to control $\alpha ^*$ and $\alpha =0.65, \mu =0.09, \gamma _{11}=0.2, \gamma _{12}=0.6, \gamma _{22}=0.2, \gamma _{21}=0.7, \varepsilon _1=0.21, \varepsilon _2=0.16, \varepsilon _3=0.23, \varepsilon _4=0.17$ to control $\beta ^*$. Figure 4a,b show the variation trends in the density of $I_1(t)$ and $I_2 (t)$ over time under different control strategies, respectively. As presented in Fig. 4, the populations of $I_1$ and $I_2$ reach the maximum when the optimal control strategy is adopted for the control variables $\alpha ^*$ and $\beta ^*$. This shows that improving the mobility and contact rate of people enlarges the spreading scope of information.

**Figure 5:**
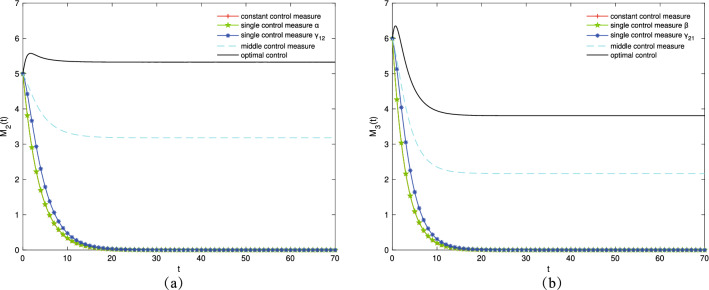
The densities of (**a**) $M_2(t)$, (**b**) $M_3(t)$ change over time under different control strategies.

Subsequently, different control strategies are adopted to increase the number of variation groups $M_2$ and $M_3$. The optimal control pairs $(\alpha ^*,{\gamma _{12}}^*)$ and $(\beta ^*,{\gamma _{21}}^*)$ are controlled, respectively. Then, let $\beta =0.65, \mu =0.05, \gamma _{11}=0.2, \gamma _{22}=0.2, \gamma _{21}=0.7, \varepsilon _1=0.17, \varepsilon _2=0.22, \varepsilon _3=0.28, \varepsilon _4=0.21$ to control optimal control pair $(\alpha ^*,{\gamma _{12}}^*)$ and $\alpha =0.76, \mu =0.09, \gamma _{11}=0.2, \gamma _{12}=0.75, \gamma _{22}=0.2, \varepsilon _1=0.23, \varepsilon _2=0.19, \varepsilon _3=0.25, \varepsilon _4=0.18$ to control optimal control pair $(\beta ^*,{\gamma _{21}}^*)$. Figure 5a,b show the variation trends in the densities of $M_2(t)$ and $M_3(t)$ over time under different control strategies, respectively. As presented in Fig. 5, $M_2(t)$ and $M_3(t)$ populations reach the maximum when the optimal control strategy is adopted for the optimal control pairs $(\alpha ^*,{\gamma _{12}}^*)$ and $(\beta ^*,{\gamma _{21}}^*)$. This shows that enhancing the education intensity and increasing the variation rate improves the variation of information.

**Figure 6:**
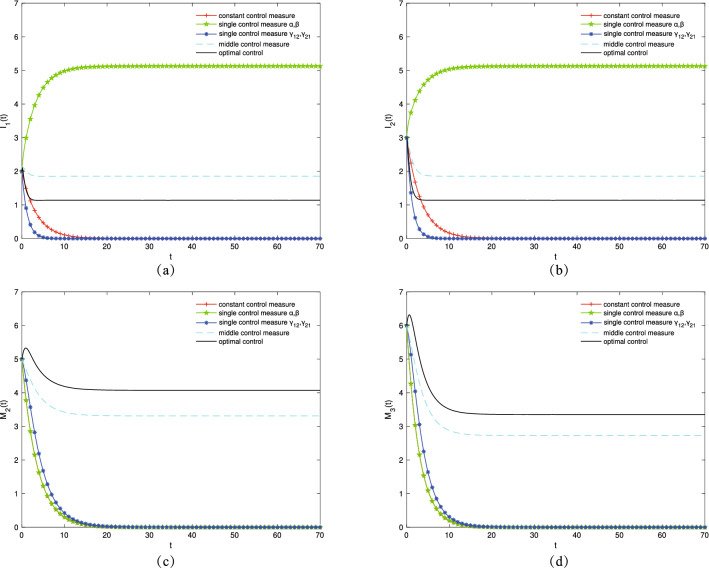
The densities of (**a**) $I_1(t)$, (**b**) $I_2(t)$, (**c**) $M_2(t)$, (**d**) $M_3(t)$ change over time under different control strategies.

Next, controlling the optimal control pairs $(\alpha ^*,\beta ^*)$ and $({\gamma 12}^*,{\gamma 21}^*)$ is adopted to increase the number of transmission groups $I_1$ and $I_2$ and variation groups $M_2$ and $M_3$ simultaneously. Let $\mu =0.09, \gamma _{11}=0.2, \gamma _{22}=0.2, \varepsilon _1=0.23, \varepsilon _2=0.19, \varepsilon _3=0.25, \varepsilon _4=0.18$. Figure 6a,b show the variation trends in the density of transmission groups $I_1(t)$ and $I_2(t)$ over time, respectively. When a single control $\alpha ^*$ and $\beta ^*$ is adopted, the population of $I_1$ and $I_2$ reaches the maximum. Figure 6c,d show the variation trends in the density of variation groups $M_2(t)$ and $M_3(t)$ over time, respectively. When an optimal control strategy is adopted, the populations of $M_2$ and $M_3$ reach the maximum.

**Figure 7:**
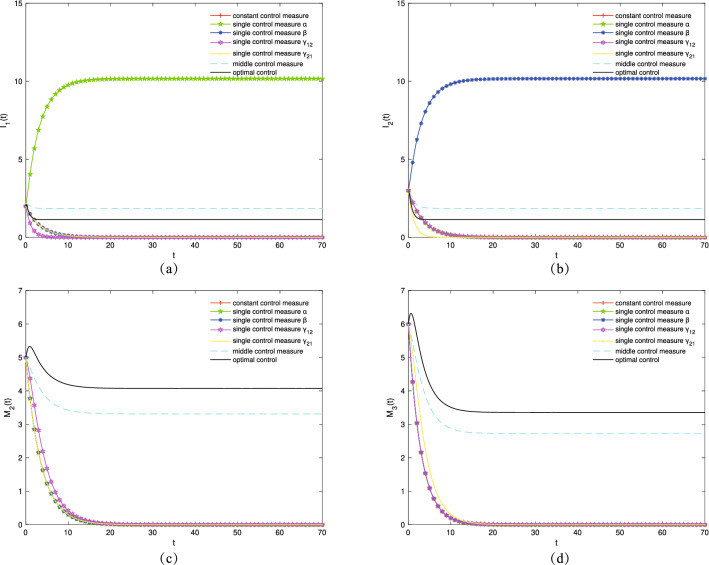
The densities of (**a**) $I_1(t)$, (**b**) $I_2(t)$, (**c**) $M_2(t)$, (**d**) $M_3(t)$ change over time under different control strategies.

Lastly, $\alpha ^*$, $\beta ^*$, ${\gamma _{12}}^*$, and ${\gamma _{21}}^*$ are controlled to increase the number of transmission groups $I_1$ and $I_2$ as well as the variation groups $M_2$ and $M_3$, respectively. Let $\mu =0.09, \gamma _{11}=0.2, \gamma _{22}=0.2, \varepsilon _1=0.23, \varepsilon _2=0.19, \varepsilon _3=0.25, \varepsilon _4=0.18$. Figure 7a,b show the variation trends in the density of transmission groups $I_1(t)$ and $I_2(t)$ over time, respectively. The $I_1$ population reaches its maximum when a single control $\alpha ^*$ is used. The $I_2$ population reaches its maximum when a single control $\beta ^*$ is used. Figure 7c,d show the variation trends in the density of variation groups $M_2(t)$ and $M_3(t)$ over time, respectively. When the optimal control strategy is adopted, the populations of $M_2$ and $M_3$ reach the maximum.

Based on the aforementioned analysis, no matter what control method is chosen, the variation group reaches the maximum when an optimal control strategy is adopted. When the transmission group is in a single control, it reaches the maximum population size under the optimal control strategy. However, when the transmission group and variation group are controlled at the same time, the populations reach the maximum only under the single control of $(\alpha ^*, \beta ^*)$.

Finally, the choice of parameters values has no established principle in the illustrations of the numerical simulations. In relevant literature on information transmission, the choice of these parameters values does not have a fixed range. Most of them are limited to positive numbers and satisfy the stability condition. In the numerical simulation, the values in other relevant literature are mentioned and the requirements of stability conditions are combined to give the numerical values of the parameters in the model. As for practical problems, determination of the specific numerical parameters is proposed, referring to the relevant professional background knowledge and investigating the actual background with reference to relevant existing literature.

## Sensitivity analysis

In order to analyze the effect of the above control variables $\alpha$ and $\beta$ on the basic reproductive number $R_0$, one need to perform the sensitivity analysis of $R_0$. It has been figure out above ${R_0} = \frac{{B\alpha ({\gamma _{22}} + {\gamma _{21}} + \mu ) + B\beta ({\gamma _{11}} + {\gamma _{12}} + \mu )}}{{\mu ({\gamma _{11}} + {\gamma _{12}} + \mu )({\gamma _{22}} + {\gamma _{21}} + \mu )}}$, thereby calculating:

60 $$\begin{aligned} & \begin{aligned} \ \frac{{\partial {R_0}}}{{\partial {\alpha }}} =\frac{{B({\gamma _{22}} + {\gamma _{21}} + \mu )}}{{\mu ({\gamma _{11}} + {\gamma _{12}} + \mu )({\gamma _{22}} + {\gamma _{21}} + \mu )}} > 0,\ \end{aligned} \end{aligned}$$

61 $$\begin{aligned} & \begin{aligned} \ \frac{{\partial {R_0}}}{{\partial {\beta }}} =\frac{{B({\gamma _{11}} + {\gamma _{12}} + \mu )}}{{\mu ({\gamma _{11}} + {\gamma _{12}} + \mu )({\gamma _{22}} + {\gamma _{21}} + \mu )}} > 0.\ \end{aligned} \end{aligned}$$

Thus it can be seen, $R_0$ is positively correlated with $\alpha$ and $\beta$. This indicates that improving the flow and contact between people can promote the transmission of information. In addition, the more information transmitters, the more information mutants.

**Figure 8:**
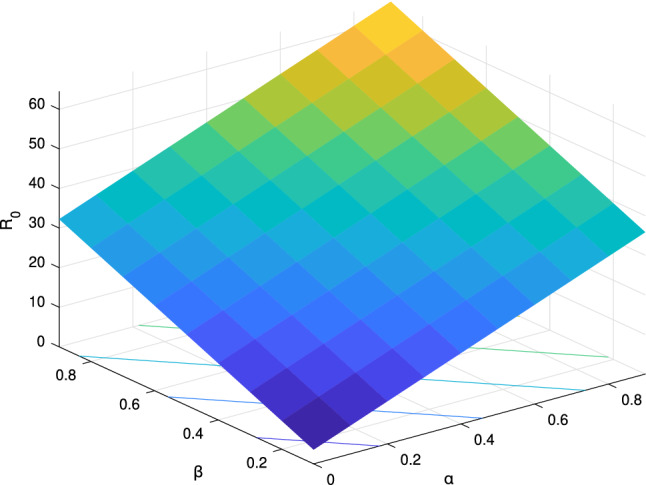
The sensitivity analysis of the basic reproduction number $R_0$.

The sensitivity analysis of $R_0$ is simulated with MATLAB R2017b in Fig. 8.

## Conclusions

In this work, we consider the influence of information cross transmission and information variation on information transmission. We construct the *S*2*I*4*MR* model of information crossing and variation, calculated the basic regeneration number of the model, analyzed the equilibrium point and stability of the model, verified the existence of the optimal control of model, and proposed the optimal control strategy of the model. Based on numerical simulations, we verify the basic theorem of the model and the effectiveness of the optimal control strategy. Finally, we analyze the sensitivity of the optimal control parameters.

The main conclusions of this work are presented below.

- The phenomenon of cross-infection and variation has been a focus of research community due to Omicron, which is the variant of *SARS-CoV-2*. It can still be applied by analogy in information transmission. As compared to previous works, the presented optimal control strategy is based on the optimal value calculated by control variables;
- By promoting the flow of people or organizing information exchange activities, it effectively improves the exchange rate of information and promotes the large-scale integration of information. Therefore, improving the natural contact rate of the two kinds of information in the crowd effectively expands the communication and fusion of information;
- By strengthening the educational guidance or putting forward encouraging multi-information application policies, it effectively promotes the cross information to evolve into new usable information by combining common advantages and ensures that the people exposed to multiple information can integrate information from different fields into widely used new information. Therefore, increasing the variation rate in the population effectively enhances the generation of new information.

The phenomenon of information crossing and variation is universal in society. On one hand, in terms of positive information, we should strengthen the information crossing and variation, so that the information after such variation can be applied in additional fields. On the other hand, in terms of negative information, we should reduce the information crossing and variation, in order to reduce its adverse impacts on the society. In the future study, we will focus on the influence of random perturbation of parameters on information transmission. In addition, as the memory effect is the most important to control and disseminating information, we will construct an information transmission model considering memory effect in the subsequent research. And extend it to the fractional derivative with a non-local kernel in future research.

## Data Availability

All data analysed during this study are included in this published article.
